# Association of Veterans Affairs Primary Care Mental Health Integration With Care Access Among Men and Women Veterans

**DOI:** 10.1001/jamanetworkopen.2020.20955

**Published:** 2020-10-20

**Authors:** Lucinda B. Leung, Lisa V. Rubenstein, Edward P. Post, Ranak B. Trivedi, Alison B. Hamilton, Jean Yoon, Erin Jaske, Elizabeth M. Yano

**Affiliations:** 1 Center for the Study of Healthcare Innovation, Implementation, and Policy, VA Greater Los Angeles Healthcare System, Los Angeles, California; 2 Division of General Internal Medicine and Health Services Research, David Geffen School of Medicine, University of California, Los Angeles; 3 Department of Health Policy and Management, Fielding School of Public Health, University of California, Los Angeles; 4 RAND Corporation, Santa Monica, California; 5 Center for Clinical Management Research, VA Ann Arbor Health Care System, Ann Arbor, Michigan; 6 Department of Medicine, University of Michigan Medical School, Ann Arbor; 7 Department of Psychiatry and Behavioral Sciences, Stanford University, Stanford, California; 8 VA Palo Alto Health Care System, Menlo Park, California; 9 Department of Psychiatry and Biobehavioral Sciences, Semel Institute for Neuroscience and Human Behavior, University of California, Los Angeles; 10 Department of General Internal Medicine, University of California, San Francisco; 11 VA Puget Sound Health Care System, Seattle, Washington

## Abstract

**Importance:**

Women veterans increasingly seek care yet continue to face barriers in the Veterans Health Administration (VA), which predominantly cares for men. Evidence-based collaborative care models can improve patient access to treatment of depression, which is experienced at higher rates by women. While the VA has implemented these care models nationally, it is not known whether access improvements occur equitably across genders in primary care.

**Objective:**

To examine whether the VA’s national Primary Care–Mental Health Integration (PC-MHI) initiative (beginning 2007) expanded realized access to mental health care similarly for men and women.

**Design, Setting, and Participants:**

This cohort study included 5 377 093 million primary care patients assigned to 396 VA clinics that provided integrated mental health services nationally between October 2013 and September 2016. Data analysis occurred between May 2017 and July 2020.

**Exposures:**

Clinic PC-MHI penetration, calculated as the proportion of clinic patients who saw an integrated specialist per fiscal year.

**Main Outcomes and Measures:**

Estimates of mean VA health care utilization (mental health, primary care, other specialty care, telephone, hospitalizations) and median total costs for men and women. Multilevel models adjusted for year, clinic, patient characteristics, and interactions between patient-defined gender and clinic PC-MHI penetration.

**Results:**

This study examined 5 377 093 veterans (448 455 [8.3%] women; 3 744 140 [69.6%] White) with a mean (SD) baseline age 62.0 (16.6) years. Each percentage-point increase in the proportion of clinic patients who saw an integrated specialist was associated with 38% fewer mental health visits per year for women (incidence rate ratio [IRR], 0.62; 95% CI, 0.60-0.65), but 39% more visits for men (IRR, 1.39; 95% CI, 1.34-1.44; *P* < .001). Both men and women had more primary care visits (men: IRR, 1.40; 95% CI, 1.36-1.45; women: IRR, 1.22; 95% CI, 1.17-1.28; *P* < .001) and total costs (men: β [SE], 2.23 [0.10]; women: β [SE], 1.24 [0.15]; *P* = .06), but women had 74% fewer hospitalizations than men related to clinics with mental health integration (IRR, 0.26; 95% CI, 0.19-0.36 vs IRR, 1.02; 95% CI, 0.83-1.24; *P* < .001).

**Conclusions and Relevance:**

While greater outpatient service use for men was observed in this study, PC-MHI was associated with a decrease in mental health specialty visits (and hospitalizations) for women veterans, potentially signifying a shift of services to primary care. With increasing patient choice for where veterans receive care, the VA must tailor medical care to the needs of rising numbers of women patients. Differences in health care utilization by gender highlight the importance of anticipating policy impacts on and tailoring services for patients in the numerical minority in the VA and other health systems.

## Key Points

**Question:**

Did the Veterans Health Administration (VA)’s national integration of mental health services into primary care beginning in 2007 improve access to care equally among men and women veterans?

**Findings:**

In this cohort study of 5.4 million veterans (including 448 455 women) who received primary care between 2013 and 2016 at VA clinics that provided integrated mental health services, mental health integration was associated with increased use of all outpatient services among men but with decreased use of services (except primary care visits) among women.

**Meaning:**

Differences in health care utilization by gender highlight the importance of anticipating policy effects and tailoring services for health system patients in the numerical minority.

## Teaser

This cohort study of Veterans Health Administration (VA) health care records examines gender differences in care usage since the national integration of mental health services into primary care for veterans.

## Introduction

Broadly undertreated, depression and anxiety are nearly twice as prevalent among women than men globally^1^ and disproportionately affect women veterans compared with men veterans in the United States.^2^ As many as half of women veterans of US wars in Iraq and Afghanistan screened positive for depression in primary care at a Veterans Health Administration (VA) facility.^3^ As in the general population, women veterans often experience treatment barriers related to pregnancy^4^ because of prioritization given to childcare and caregiving,^5^ with the added complications of experiences of military sexual trauma^6,7^ and intimate partner violence.^8^ Compared with civilian women, suicide completion rates are 2.5 times higher among women veterans, and have continued to increase 85% in recent years.^9^

Growing numbers of women veterans (estimated to increase from 10.0% to 14.3% of veterans by 2033)^10^ seek medical treatment in the VA, but they remain a numerical minority navigating a health care system that predominantly cares for men. One in 4 women veterans have reported harassment from men in VA facilities, an experience that has been associated with their delaying or missing care.^11^

During the past decade, the VA invested resources to improve mental health care access through the national Primary Care–Mental Health Integration (PC-MHI) initiative,^12^ but we do not know if the improvements similarly benefitted men and women veterans. The PC-MHI initiative generally targets increased access to effective depression and other mental health treatments for veterans. Through the care models implemented by the PC-MHI, primary care clinicians, mental health specialists, and/or care managers jointly manage mild-to-moderate psychiatric conditions directly in primary care settings^13^ and redirect severe conditions for intensive specialty mental health treatment. This may mean reducing reliance on specialty mental health services when it is not necessary and/or increasing delivery of collocated services for patients who decline referrals because of stigma or other concerns.

Few studies have examined integrated mental health care and its effects on women specifically,^4^ and few health care professionals have attempted to tailor collaborative care services for women veterans.^14^ The evidence is mixed regarding whether men and women experience different outcomes from integrated care for depression and anxiety, ranging from having no gender-related associations to having better clinical outcomes (eg, depression remission, greater quality of life) among women compared with men.^15,16,17^ In the early years of the PC-MHI initiative, larger proportions of women veterans than men veterans with a psychiatric diagnosis used PC-MHI services (ie, in a primary care setting) than specialty mental health clinic services (8.6% vs 7.7%).^18^ Less is known about gender-associated PC-MHI outcomes more recently, given that women veterans have substantially increased in number and PC-MHI is newly supported by the VA’s patient-centered medical home initiative (PACT).

Being a numerical minority in the VA, with often separate care delivery structures, women likely experience differences in mental health care access.^19,20^ In a 2019 study, we found that the VA’s investment in PC-MHI models has been associated with improved veteran access to mental health services as measured by higher utilization^12^ but do not know whether there are differences by gender. Understanding whether changes in health care utilization associated with PC-MHI differ by gender may help to facilitate the VA’s provision of timely, gender-sensitive health care^21^ and subsequently better overall care experience among women veterans.^22^

This study examined the associations of VA’s national PC-MHI initiative, approximated by the proportion of clinic patients who saw integrated mental health specialists, with health care utilization and total costs for women vs men. Given that psychiatric illnesses targeted by this initiative are more prevalent in women, we hypothesized that PC-MHI may be associated with greater realized access to mental health care, demonstrated by more mental health visits in women than in men. Given how differently men and women experience VA care, we hypothesized that PC-MHI’s associations with health care use (eg, in primary care, other specialty care, via telephone, and hospitalizations) and total costs may also differ by gender.

## Methods

### Study Design and Cohort

Because the evaluation efforts were part of an ongoing quality improvement effort at the VA, this study was deemed to be nonhuman subjects research and therefore exempt from informed consent requirements by the institutional review board. In our retrospective longitudinal cohort study of data recorded between October 1, 2013, and September 30, 2016, 5 377 093 patients were assigned and received care at 1 of 396 VA primary care clinics required to provide onsite PC-MHI services (153 hospital-based, 243 community-based) per Patient Centered Management Module databases. Each clinic provided general, women’s health, and/or geriatrics primary care to 5000 or more veterans annually. While the VA mandated onsite PC-MHI services, the organization of services (eg, staffing arrangements) can vary from clinic to clinic.^23^ In this follow-up study,^12^ we stratified patients by gender, 4 928 638 men (12 466 356 person-years) and 448 455 women veterans (1 126 448 person-years), to test whether PC-MHI initiative associations differed by gender. This study followed the Strengthening the Reporting of Observational Studies in Epidemiology (STROBE) reporting guideline.

### Measures

#### Primary Outcomes

We used nationally designated electronic encounter codes from the VA’s Corporate Data Warehouse (CDW) and subdivided visits as follows: mental health (ie, any visit to a mental health specialist, including at clinics with integrated PC-MHI services), primary care, other (ie, non–mental health) specialty care, telephone, and hospitalization (psychiatric and nonpsychiatric). Then, we estimated total VA health care costs by multiplying health care use and unit cost estimates from the VA’s Decision Support System files, which did not include VA-sponsored care from non-VA clinicians.^24^ We reported relative measures of the association with each percentage-point increase in clinic PC-MHI penetration and mean numbers of health care visits and costs per person per year.

#### Main Independent Variable

We obtained the annual PC-MHI penetration rate, a performance indicator of PC-MHI’s reach into a clinic’s primary care patient population, from the VA’s Support Service Center. It is calculated as the proportion of primary care patients who saw integrated mental health specialists in each clinic annually.^12^

#### Covariates

The study controlled for utilization-related patient and clinic characteristics available from VA’s CDW, National Patient Care Database, Area Resource File, and Planning Systems Support Group. Patient covariates included age, gender (as defined by the patient), race/ethnicity (ie, non-Hispanic White, non-Hispanic Black, Hispanic, other, or missing), marital status, income proxies (because patients may be eligible for VA care based on a service-connected disability or required clinic copayment), and distance from home address to home clinic. We used *International Classification of Diseases, Ninth Revision *(*ICD*-*9*) and *International Statistical Classification of Diseases and Related Health Problems, Tenth Revision *(*ICD*-*10*) diagnostic codes (eAppendix 1 in the Supplement) found in outpatient and inpatient visits for depression, anxiety, posttraumatic stress disorder (PTSD), alcohol and substance use disorders, serious mental illness (ie, schizophrenia, bipolar disorder), and homelessness in each study year. We used the comorbidity score formulated in Gagne et al,^25^ which combined medical conditions in the Charlson and Elixhauser measures, and we subdivided scores into 3 levels of severity for each patient in each year. To identify any gender-moderating associations, we constructed an interaction term between gender and the clinic PC-MHI penetration rate.

We also examined clinic characteristics, including hospital-based vs community-based, rural vs nonrural location, and number of assigned primary care patients (as a proxy for clinic size) in descriptive analyses. To account for the extent of patient-centered medical home implementation in each VA clinic, we adjusted for the PACT Implementation Progress Index (PI^2^), which is an established measure of 8 core patient-centered medical home components (eg, access, care coordination).^26^

### Statistical Analysis

In descriptive statistics, we compared baseline patient-level characteristics at baseline by gender using *t* and χ^2^ tests. Because the service delivery structure may differ between men and women veterans, we additionally compared clinic-level characteristics. Furthermore, we calculated mean numbers (with SDs) of medical visits per patient from 2013 to 2016, comparing male and female VA primary care patients using *t* tests.

Using multilevel regression models, we estimated the association of clinic PC-MHI penetration with health care utilization and cost outcomes for all study patients after adjusting for year and clinic fixed effects, PI^2^, gender × clinic PC-MHI penetration interaction, and utilization-related patient characteristics. We included year and clinic fixed effects to account for secular trends and invariant clinic characteristics. Patient random effects were included to account for the possibility of patients having multiple nonindependent observations during the 3 study years. Given the count distributions of our utilization outcomes, we used and reported incidence rate ratios (IRRs) and 95% CIs from multilevel Poisson regressions in adjusted models. Sensitivity analyses were conducted using negative binomial regression and zero-inflated models for our utilization outcomes. Because our health care costs had a skewed distribution, we used log-transformed costs in our multilevel linear regression models and reported coefficients (or geometric average costs) and SEs. We determined significance of gender differences based on the gender × PC-MHI interaction. Because PC-MHI penetration has been observed to differ by clinic type,^12^ we stratified all models by hospital-based and community-based clinics.

For all models, we determined significance by using a 2-tailed α = .05. Data were analyzed in SAS version 9.4 (SAS Institute).

## Results

### Patient and Clinic Characteristics

In our study of 5 377 093 million veterans (mean [SD] baseline age, 62.0 [16.6] years; 448 455 [8.3%] women; 3 744 140 [69.6%] White), men and women veterans differed significantly in several baseline characteristics (Table). Women veterans were younger compared with men (mean [SD] age, 48.7 [15.1] years vs 63.1 [16.1] years; *P* < .001) and less likely to be married (128 625 [35.8%] vs 2 305 236 [55.3%]; *P* = .04). While prevalence rates were substantially higher than in the general population, we again observed approximately twice the rate of diagnosed depression in women compared with men (98 788 [28%] vs 670 926 [16%]; *P* = .04). Women were treated more often in primary care clinics that had higher clinic PC-MHI penetration rates compared with men (median [interquartile range {IQR}], 6.5% [4.5%-8.5%] vs 6.2% [4.2%-8.2%]; *P* < .001).

**Table.** VA Primary Care Patient Characteristics by Gender for Fiscal Year 2014

Characteristics	Patients, No. (%)		
	Men (n = 4 928 638)	Women (n = 448 455)	
Age, y^a^			
18-44	595 678 (14.4)	162 360 (40.8)	
45-54	463 870 (11.2)	81 129 (24.2)	
55-64	855 638 (20.7)	75 287 (22.5)	
65-74	1 252 078 (30.2)	24 127 (7.2)	
75-84	623 456 (15.1)	9051 (2.7)	
≥85	349 759 (8.5)	8542 (2.5)	
Race/ethnicity			
Non-Hispanic White	2 983 546 (71.5)	197 011 (54.9)	
Non-Hispanic Black	719 603 (17.3)	106 339 (29.6)	
Hispanic	283 969 (6.8)	27 724 (7.7)	
Other^b^	183 946 (4.4)	27 833 (7.8)	
Marital status^c^			
Single	507 524 (12.2)	79 469 (22.1)	
Married	2 305 236 (55.3)	128 625 (35.8)	
Divorced, separated, or widowed	1 358 303 (32.6)	150 813 (42.0)	
VA health benefits copayments			
Exempt from copayments	3 360 995 (91.1)	295 959 (91.5)	
Any copayment required	293 410 (8.0)	25 624 (7.9)	
Missing data	32 094 (0.9)	1956 (0.6)	
Service connectedness, %			
0	2 081 930 (49.9)	147 220 (41.0)	
1-50	936 415 (22.5)	84 567 (25.0)	
51-100	1 152 719 (27.6)	122 120 (34.0)	
Unhomed	131 477 (3.2)	9025 (2.5)	
Gagne comorbidity score^d^			
Low, bottom 25%	820 753 (19.7)	76 240 (21.2)	
Intermediate, middle 25%-75%	2 114 517 (50.7)	176 059 (49.1)	
High-risk, top 25%	1 235 794 (29.6)	106 608 (29.7)	
Mental health diagnoses			
Depression^c^	670 926 (16.2)		98 788 (27.5)
Posttraumatic stress disorder	475 584 (11.4)		53 668 (15.0)
Substance and alcohol use disorder	465 777 (11.2)		26 392 (7.4)
Anxiety	300 139 (7.2)		50 575 (14.1)
Schizophrenia	66 205 (1.6)		5638 (1.6)
Bipolar disorder	87 955 (2.1)		18 293 (5.1)
Average distance traveled to clinic, median (IQR), miles^a^	11 (6-21)		11 (6-18)
VA health care use, mean (SD)			
Mental health visits^a^	2.9 (10.9)		4.9 (13.1)
Primary care visits^a^	3.3 (3.9)		4.1 (4.5)
Other specialty care visits^a^	1.1 (2.8)		1.0 (2.6)
Telephone visits^a^	1.6 (3.1)	2.0 (3.3)	
Hospitalizations^a^	0.2 (0.7)	0.1 (0.5)	
Total cost, median (IQR), $^a^	2535.06 (875.88-6837.18)	3363.63 (1204.96-8091.22)	

Differences between gender were tested for significance using χ^2^ tests or *t* tests.

^a^ *P* < .001.

^b^ Other included Asian, multiracial, Alaskan native, American Indian, Pacific Islander, and Native Hawaiian.

^c^ *P* < .05.

^d^ Comorbidity score (formulated in Gagne et al^25^) combines medical conditions in the Charlson and Elixhauser measures; we subdivided scores into 3 levels of severity for each patient in each year.

### Unadjusted Analyses

In the baseline year, health care utilization and cost differed significantly between women and men veterans (Table). Among women, mean (SD) number of visits for mental health was 1.8 times greater (4.9 [13.1] vs 2.9 [10.9]; *P* < .001), the number of primary care visits was 1.2 times greater (4.1 [4.5] vs 3.3 [3.9]; *P* < .001), and the number of telephone care calls was approximately 24% greater (2.0 [3.3] vs 1.6 [3.1]; *P* < .001) compared with men. Conversely, women visited other specialists 2% less (mean [SD], 1.0 [2.6] vs 1.1 [2.8]; *P* < .001) and were hospitalized 17% less (mean [SD], 0.1 [0.5] vs 0.2 [0.7]; *P* < .001) than men. Altogether, total costs were $847.90 higher for women (median [IQR], $3382.96 [$1200.50-$8122.10] vs $2535.06 [$875.88-$6837.18]; *P* < .001) than for men.

### Adjusted Analyses

In fully adjusted models, we identified significant gender differences in the association of VA’s national PC-MHI initiative with VA health care utilization (Figure). With every 1 percentage-point increase in primary care patients who saw integrated specialists (ie, clinic PC-MHI penetration), we observed an associated 38% decrease in the mean number of mental health visits per patient per year among women (incidence rate ratio [IRR], 0.62; 95% CI, 0.60-0.65), but a 39% increase among men (IRR, 1.39; 95% CI, 1.34-1.44; *P* < .001). Women were less likely than men to visit non–mental health specialists (IRR, 0.52; 95% CI, 0.48-0.57 vs IRR, 0.87; CI, 0.82-0.92; *P* < .001) and be hospitalized (IRR, 0.26; 95% CI, 0.19-0.36 vs IRR, 1.02; 95% CI, 0.83-1.24; *P* < .001) with each percentage-point increase in clinic PC-MHI penetration. We similarly observed larger increases in primary care visits among women than men (women: IRR, 1.22; 95% CI, 1.17-1.28; men: IRR, 1.40; 95% CI, 1.36-1.45; *P* < .001) with each percentage-point increase in clinic PC-MHI penetration. There were no significant gender differences in telephone care (IRR, 1.22; 95% CI, 1.14-1.31 vs IRR, 1.28; 95% CI, 1.22-1.35; *P* = .08), nor were there significant differences in total costs (women: β [SE], 1.24 [0.15]; men: β [SE], 2.23 [0.10]; *P* = .06) associated with clinic PC-MHI penetration.

**Figure.**
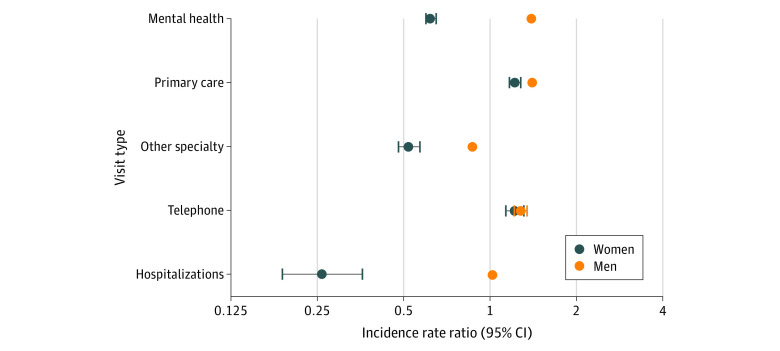
Associations of VA Health Care Use Among Men and Women Veterans With Increase in Penetration of Integrated Mental Health Services in Clinics Points indicate incidence rate ratios (IRR), and error bars indicate 95% CIs. IRRs are interpreted as percentage change in average health services use per person per year, relative to each percentage-point increase in clinic Primary Care–Mental Health Integration (PC-MHI) penetration. Multilevel Poisson regression models adjusted for year, clinic site, VA patient-centered medical home initiative (PACT) implementation Progress Index (PI^2^), and patient characteristics, which included age, gender, race/ethnicity, marital status, VA means test, service connectedness, Gagne score category, homelessness, distance from home to clinic, depression, anxiety, posttraumatic stress disorder, substance use disorder, serious mental illness (ie, schizophrenia, bipolar disorder). *P* < .001 for the interaction between clinic PC-MHI penetration and gender, which indicates significant associations of PC-MHI with health services use by gender; treatment by telephone is excluded from this interaction.

### Additional Analyses

When comparing women treated in hospital-based clinics with community-based clinics, we observed that each 1% increase in PC-MHI penetration was associated with greater reductions in the mean number of mental health visits with a persistently significant gender × PC-MHI interaction (hospital-based clinics: IRR, 0.81; 95% CI, 0.76-0.86; community-based clinics: IRR, 0.55; 95% CI, 0.52-0.59; *P* < .001). Observed differences in associations of PC-MHI with health care use (eg, in primary care, non–mental health specialty care, and hospitalizations) by gender were no longer significant when we restricted our analyses to only those treated in community-based clinics.

## Discussion

Associations of the VA’s national PC-MHI initiative and health care utilization significantly differed between men and women veterans, and it remains unclear whether observed gender differences are desirable. Integrated mental health treatment in primary care was associated with fewer specialist visits (including for mental health) and hospitalizations for women veterans but not for men. Our hypothesis that treatment in clinics where greater proportions of primary care patients saw a PC-MHI clinician would be associated with greater mental health care utilization among women was not supported—in stark contrast with the strong positive association seen in men. Similar to men, women veterans were found to have more primary care visits related to integrated mental health services. We posit that while PC-MHI may engage men to address unmet needs through both mental health and primary care, it may alternatively engage women to seek treatment for similar issues through primary care, allowing the same clinician to meet all their health needs during 1 visit. Consistent with collaborative care principles,^27^ primary care health professionals may be empowered by PC-MHI support (eg, additional training and informal or so-called curbside consultation) to independently treat women veterans with psychiatric illness. Prior research has demonstrated that shifting specialty mental health care to PC-MHI–delivered services in primary care was not observed to negatively affect quality of care.^23^ Future studies should address whether observed health care utilization changes reflect where women veterans prefer to receive mental health care when given the option of having most needs met in primary care.

Gender differences in PC-MHI associations also were found on other types of health care visits. Among men, we observed that increasing clinic PC-MHI penetration was associated with more frequent utilization of all outpatient services except non–mental health specialty care. We noted greater use of primary care by women as well, but found increasing clinic PC-MHI penetration to be associated with lower specialty care use and lower hospitalization rates compared with men. Among women veterans, fewer non–mental health specialty visits and hospitalizations may indicate better control of physical health conditions when mental health problems are well-managed by primary care that is supported by PC-MHI programs. While health care utilization patterns differed, we found no gender differences in PC-MHI–associated cost outcomes. Additional research is needed to explore whether increasing clinic PC-MHI penetration is associated with improvements in women veterans’ health outcomes, which may account for fewer non–mental health specialty care visits and hospitalizations.

Our large, national examination of women veterans supports prior research in the VA and in the general population documenting gender differences in mental health and care delivery preferences. Women veterans have again been found to have twice the rates of depression and anxiety^1,2^ and use more mental health and primary care services than men.^28^ Women veterans are known to prioritize various competing demands (ie, childrearing) over medical care^5^ and prefer collocating medical (and mental health) services,^21,29^ which supports our observed shift in mental health service delivery within PC-MHI–supported primary care. Altogether, our findings concurrently suggest that women more readily access mental health care than men and may prefer such care to be delivered in primary care (or at least in the same convenient location).

Other organizational characteristics, such as the creation of dedicated women’s health clinics to deliver primary care and gender-specific services, likely also contribute to observed gender differences in health care utilization. Currently, women veterans may receive care in women’s health clinics (often with colocated women’s mental health specialists) or in the same clinic space where men are seen (with PC-MHI services offered).^19,20^ In this study, women veterans were more likely than men to receive care in hospital-based clinics, where average PC-MHI penetration rates are higher^12^ and where women-only treatment settings are commonly located.^19,20^ Findings from our sensitivity analyses on hospital-based vs community-based clinics suggested that clinic type may contribute to gender differences observed among veteran health care utilization patterns. Therefore, other organizational factors (eg, women’s mental health expertise, clinician and staff gender-sensitivity, and the variability of PC-MHI implementation^23^) should be examined to optimally arrange VA care for women veterans.^10^ Because women veterans, like women in the general population, are more likely to have conditions targeted for collaborative care intervention, we need to understand how they access mental health services differently than men and tailor integrated services to their needs.

### Limitations

This study had several limitations. First, observed health care utilization patterns did not examine other care access or patient outcomes, meaning these patterns may demonstrate women veterans’ preference for treatment location, approximate their access to medical care, or both. These findings indicate associations and do not permit causal inference. Second, our analyses did not distinguish between VA women’s health clinics and general primary care, control for several factors related to care integration (eg, mental health care staffing), or include emergency visits, all of which may affect health care utilization and merit additional research. Third, our VA administrative data sources are from 5 years ago and may be subject to coding inaccuracies. For example, our study period overlapped with the transition from *ICD*-*9* to *ICD*-*10* diagnostic codes, during which lower coding of certain mental health conditions (eg, alcohol use disorders) has been documented.^30^ Fourth, our analyses did not include medical care received in outside health care systems and thus do not generalize well to non-VA primary care use by women veterans or civilian women. Women veterans who use VA care report worse overall health, lower income, and less often have health insurance than women veterans who do not use VA care^31,32^; as such, these findings may inform health care delivery and policy for this high-need population.

## Conclusions

In this study, as in the general population, women veterans had high rates of depression and anxiety, which could be effectively treated through improved integration of primary care and mental health services. Primary care clinics where a greater proportion of patients see integrated mental health specialists exhibit different mental health utilization patterns among men (increased use) and women (decreased use) veterans, compared with clinics where a lower proportion see integrated clinicians. While integrated care models may increase engagement in mental health care for men, it may also be shifting mental health care (as well as non–mental health specialty care and hospitalizations) to primary care for women veterans. This may reflect women veterans’ preference for receiving all care in 1 location, the ongoing barriers to navigating mental health and specialty care in a traditionally male-dominated health care system, or a combination of both.

Gender differences in health care utilization associated with the VA’s national PC-MHI highlight the importance of anticipating differences and tailoring services for at-risk patients in health systems. While more research is needed to fully interpret how these findings change our perspective on how women’s health care should be organized in health systems like the VA, integrated care models appear to offer women veterans greater choice on where to receive mental health treatment—in primary care or specialty settings. With increasing patient choice in where to receive care, the VA and other health systems should consider additional research to inform improvement in mental health care access and to anticipate the differential effects of national or systemwide policies by gender and on other patient minority groups.

## Notes

Supplement. **eAppendix.** *ICD*-*9* and *ICD*-*10* Diagnostic Codes

## References

[1] Baxter AJ, Scott KM, Ferrari AJ, Norman RE, Vos T, Whiteford HA Challenging the myth of an “epidemic” of common mental disorders: trends in the global prevalence of anxiety and depression between 1990 and 2010. Depress Anxiety. 2014;31(6):506-516. doi:10.1002/da.22230 24448889

[2] Maguen S, Ren L, Bosch JO, Marmar CR, Seal KH Gender differences in mental health diagnoses among Iraq and Afghanistan veterans enrolled in veterans affairs health care. Am J Public Health. 2010;100(12):2450-2456. doi:10.2105/AJPH.2009.166165 20966380 PMC2978175

[3] Haskell SG, Gordon KS, Mattocks K, Gender differences in rates of depression, PTSD, pain, obesity, and military sexual trauma among Connecticut war veterans of Iraq and Afghanistan. J Womens Health (Larchmt). 2010;19(2):267-271. doi:10.1089/jwh.2008.1262 20109115 PMC3052274

[4] Hamilton AB, Farmer MM, Moin T, Enhancing Mental and Physical Health of Women through Engagement and Retention (EMPOWER): a protocol for a program of research. Implement Sci. 2017;12(1):127. doi:10.1186/s13012-017-0658-9 29116022 PMC5678767

[5] Washington DL, Bean-Mayberry B, Riopelle D, Yano EM Access to care for women veterans: delayed healthcare and unmet need. J Gen Intern Med. 2011;26(suppl 2):655-661. doi:10.1007/s11606-011-1772-z 21989618 PMC3191223

[6] Kimerling R, Street AE, Pavao J, Military-related sexual trauma among Veterans Health Administration patients returning from Afghanistan and Iraq. Am J Public Health. 2010;100(8):1409-1412. doi:10.2105/AJPH.2009.171793 20558808 PMC2901286

[7] Kimerling R, Gima K, Smith MW, Street A, Frayne S The Veterans Health Administration and military sexual trauma. Am J Public Health. 2007;97(12):2160-2166. doi:10.2105/AJPH.2006.092999 17971558 PMC2089100

[8] Dichter ME, Cerulli C, Bossarte RM Intimate partner violence victimization among women veterans and associated heart health risks. Womens Health Issues. 2011;21(4)(suppl):S190-S194. doi:10.1016/j.whi.2011.04.008 21724140

[9] Price J Battling depression and suicide among female veterans. *All Things Considered* National Public Radio May 29, 2018 Accessed September 2, 2020. https://www.npr.org/2018/05/29/614011243/battling-depression-and-suicide-among-female-veterans

[10] Yano EM, Hayes P, Wright S, Integration of women veterans into VA quality improvement research efforts: what researchers need to know. J Gen Intern Med. 2010;25(suppl 1):56-61. doi:10.1007/s11606-009-1116-4 20077153 PMC2806960

[11] Klap R, Darling JE, Hamilton AB, Prevalence of stranger harassment of women veterans at veterans affairs medical centers and impacts on delayed and missed care. Womens Health Issues. 2019;29(2):107-115. doi:10.1016/j.whi.2018.12.002 30686577

[12] Leung LB, Rubenstein LV, Yoon J, Veterans Health Administration investments in primary care and mental health integration improved care access. Health Aff (Millwood). 2019;38(8):1281-1288. doi:10.1377/hlthaff.2019.00270 31381382

[13] Post EP, Metzger M, Dumas P, Lehmann L Integrating mental health into primary care within the Veterans Health Administration. Fam Syst Health. 2010;28(2):83-90. doi:10.1037/a0020130 20695668

[14] Huang H, Tabb KM, Cerimele JM, Ahmed N, Bhat A, Kester R Collaborative care for women with depression: a systematic review. Psychosomatics. 2017;58(1):11-18. doi:10.1016/j.psym.2016.09.002 27842779

[15] Bauer AM, Azzone V, Alexander L, Goldman HH, Unützer J, Frank RG Are patient characteristics associated with quality of depression care and outcomes in collaborative care programs for depression? Gen Hosp Psychiatry. 2012;34(1):1-8. doi:10.1016/j.genhosppsych.2011.08.019 22018769 PMC3253908

[16] Grubbs KM, Cheney AM, Fortney JC, The role of gender in moderating treatment outcome in collaborative care for anxiety. Psychiatr Serv. 2015;66(3):265-271. doi:10.1176/appi.ps.201400049 25727114 PMC4453769

[17] Sherbourne CD, Weiss R, Duan N, Bird CE, Wells KB Do the effects of quality improvement for depression care differ for men and women? Results of a group-level randomized controlled trial. Med Care. 2004;42(12):1186-1193. doi:10.1097/00005650-200412000-00005 15550798

[18] Johnson-Lawrence VD, Szymanski BR, Zivin K, McCarthy JF, Valenstein M, Pfeiffer PN Primary care-mental health integration programs in the veterans affairs health system serve a different patient population than specialty mental health clinics. Prim Care Companion CNS Disord. 2012;14(3):PCC.11m01286. doi:10.4088/PCC.11m01286 23106026 PMC3466035

[19] Yano EM, Haskell S, Hayes P Delivery of gender-sensitive comprehensive primary care to women veterans: implications for VA Patient Aligned Care Teams. J Gen Intern Med. 2014;29(suppl 2):S703-S707. doi:10.1007/s11606-013-2699-3 24715395 PMC4070234

[20] Oishi SM, Rose DE, Washington DL, MacGregor C, Bean-Mayberry B, Yano EM National variations in VA mental health care for women veterans. Womens Health Issues. 2011;21(4)(suppl):S130-S137. doi:10.1016/j.whi.2011.04.029 21724132

[21] Kimerling R, Bastian LA, Bean-Mayberry BA, Patient-centered mental health care for female veterans. Psychiatr Serv. 2015;66(2):155-162. doi:10.1176/appi.ps.201300551 25642611 PMC4776740

[22] Brunner J, Schweizer CA, Canelo IA, Leung LB, Strauss JL, Yano EM Timely access to mental health care among women veterans. Psychol Serv. 2018;16(3):498-503. doi:10.1037/ser0000226 29620391 PMC6773248

[23] Leung LB, Rose D, Stockdale S, Regional adoption of primary care-mental health integration in Veterans Health Administration patient-centered medical homes. J Healthc Qual. 2019;41(5):297-305. doi:10.1097/JHQ.0000000000000206 31135605

[24] Hebert PL, Liu CF, Wong ES, Patient-centered medical home initiative produced modest economic results for Veterans Health Administration, 2010-12. Health Aff (Millwood). 2014;33(6):980-987. doi:10.1377/hlthaff.2013.0893 24889947

[25] Gagne JJ, Glynn RJ, Avorn J, Levin R, Schneeweiss S A combined comorbidity score predicted mortality in elderly patients better than existing scores. J Clin Epidemiol. 2011;64(7):749-759. doi:10.1016/j.jclinepi.2010.10.004 21208778 PMC3100405

[26] Nelson KM, Helfrich C, Sun H, Implementation of the patient-centered medical home in the Veterans Health Administration: associations with patient satisfaction, quality of care, staff burnout, and hospital and emergency department use. JAMA Intern Med. 2014;174(8):1350-1358. doi:10.1001/jamainternmed.2014.2488 25055197

[27] Archer J, Bower P, Gilbody S, Collaborative care for depression and anxiety problems. Cochrane Database Syst Rev. 2012;10:CD006525. doi:10.1002/14651858.CD006525.pub2 23076925 PMC11627142

[28] Davis TD, Campbell DG, Bonner LM, Women veterans with depression in Veterans Health Administration primary care: an assessment of needs and preferences. Womens Health Issues. 2016;26(6):656-666. doi:10.1016/j.whi.2016.08.001 27697494

[29] Washington DL, Bean-Mayberry B, Hamilton AB, Cordasco KM, Yano EM Women veterans’ healthcare delivery preferences and use by military service era: findings from the National Survey of Women Veterans. J Gen Intern Med. 2013;28(suppl 2):S571-S576. doi:10.1007/s11606-012-2323-y 23807067 PMC3695266

[30] Yoon J, Chow A Comparing chronic condition rates using *ICD*-*9* and *ICD*-*10* in VA patients FY2014-2016. BMC Health Serv Res. 2017;17(1):572. doi:10.1186/s12913-017-2504-9 28818082 PMC5561575

[31] Hamilton AB, Frayne SM, Cordasco KM, Washington DL Factors related to attrition from VA healthcare use: findings from the National Survey of Women Veterans. J Gen Intern Med. 2013;28(suppl 2):S510-S516. doi:10.1007/s11606-013-2347-y 23807058 PMC3695263

[32] Mattocks KM, Yano EM, Brown A, Casares J, Bastian L Examining women veterans’ experiences, perceptions, and challenges with the veterans choice program. Med Care. 2018;56(7):557-560. doi:10.1097/MLR.0000000000000933 29768310

