# Supplementary material for: Association of Veterans Affairs Primary Care Mental Health Integration With Care Access Among Men and Women Veterans
Source: JAMA Netw Open. 2020 Oct 20;3(10):e2020955. doi: 10.1001/jamanetworkopen.2020.20955 (PMC7576407; doi:10.1001/jamanetworkopen.2020.20955)
Supplement: Supplement. — eAppendix. ICD-9 and ICD-10 Diagnostic Codes [file jamanetwopen-e2020955-s001.pdf]

Supplemental Online Content

Leung LB, Rubenstein LV, Post EP, et al. Association of Veterans Affairs primary care mental health integration with care access among men and women veterans. *JAMA Netw Open*. 2020;3(10):e2020955. doi:10.1001/jamanetworkopen.2020.20955

eAppendix. *ICD-9* and *ICD-10* Diagnostic Codes

This supplemental material has been provided by the authors to give readers additional information about their work.

© 2020 Leung LB et al. *JAMA Network Open.*

Appendix. *ICD-9* and *ICD-10* Diagnostic Codes

ICD 9 Codes	
PTSD	
309.81	POSTTRAUMATIC STRESS DISORDER
309.81	PROLONGED POSTTRAUMATIC STRESS DISORDER
DEPRESSION	
293.83	MOOD DISORDER IN CONDITIONS CLASSIFIED ELSEWHERE
293.83	ORGANIC AFFECTIVE SYNDROME
296.2	MAJOR DEPRESSIVE AFFECTIVE DISORDER, SINGLE EPISODE, UNSPECIFIED DEGREE
296.21	MAJOR DEPRESSIVE AFFECTIVE DISORDER, SINGLE EPISODE, MILD DEGREE
296.22	MAJOR DEPRESSIVE AFFECTIVE DISORDER, SINGLE EPISODE, MODERATE DEGREE
296.23	MAJOR DEPRESSIVE AFFECTIVE DISORDER, SINGLE EPISODE, SEVERE DEGREE, WITHOUT MENTION OF PSYCHOTIC BEHAVIOR
296.24	MAJOR DEPRESSIVE AFFECTIVE DISORDER, SINGLE EPISODE, SEVERE DEGREE, SPECIFIED AS WITH PSYCHOTIC BEHAVIOR
296.25	MAJOR DEPRESSIVE AFFECTIVE DISORDER, SINGLE EPISODE, IN PARTIAL OR UNSPECIFIED REMISSION
296.26	MAJOR DEPRESSIVE AFFECTIVE DISORDER, SINGLE EPISODE, IN FULL REMISSION
296.3	MAJOR DEPRESSIVE AFFECTIVE DISORDER, RECURRENT EPISODE, UNSPECIFIED DEGREE
296.31	MAJOR DEPRESSIVE AFFECTIVE DISORDER, RECURRENT EPISODE, MILD DEGREE
296.32	MAJOR DEPRESSIVE AFFECTIVE DISORDER, RECURRENT EPISODE, MODERATE DEGREE
296.33	MAJOR DEPRESSIVE AFFECTIVE DISORDER, RECURRENT EPISODE, SEVERE DEGREE, WITHOUT MENTION OF PSYCHOTIC BEHAVIOR
296.34	MAJOR DEPRESSIVE AFFECTIVE DISORDER, RECURRENT EPISODE, SEVERE DEGREE, SPECIFIED AS WITH PSYCHOTIC BEHAVIOR
296.35	MAJOR DEPRESSIVE AFFECTIVE DISORDER, RECURRENT EPISODE, IN PARTIAL OR UNSPECIFIED REMISSION
296.36	MAJOR DEPRESSIVE AFFECTIVE DISORDER, RECURRENT EPISODE, IN FULL REMISSION
296.9	UNSPECIFIED AFFECTIVE PSYCHOSIS
296.9	UNSPECIFIED EPISODIC MOOD DISORDER
296.99	OTHER SPECIFIED AFFECTIVE PSYCHOSES
296.99	OTHER SPECIFIED EPISODIC MOOD DISORDER
300.4	DYSTHYMIC DISORDER
300.4	NEUROTIC DEPRESSION
309.1	ADJUSTMENT REACTION WITH PROLONGED DEPRESSIVE REACTION
311	DEPRESSIVE DISORDER, NOT ELSEWHERE CLASSIFIED
SUD	
303.9	OTHER AND UNSPECIFIED ALCOHOL DEPENDENCE, UNSPECIFIED DRINKING BEHAVIOR
304.1	SEDATIVE, HYPNOTIC OR ANXIOLYTIC DEPENDENCE, UNSPECIFIED
304.22	COCAINE DEPENDENCE, EPISODIC USE
304.42	AMPHETAMINE AND OTHER PSYCHOSTIMULANT DEPENDENCE, EPISODIC USE
304.61	OTHER SPECIFIED DRUG DEPENDENCE, CONTINUOUS USE
305.22	CANNABIS ABUSE, EPISODIC USE
305.3	HALLUCINOGEN ABUSE UNSPECIFIED

© 2020 Leung LB et al. *JAMA Network Open.*

305.32	HALLUCINOGEN ABUSE, EPISODIC USE
305.4	BARBITURATE AND SIMILARLY ACTING SEDATIVE OR HYPNOTIC ABUSE UNSPECIFIED
305.41	SEDATIVE, HYPNOTIC OR ANXIOLYTIC ABUSE, CONTINUOUS
305.42	SEDATIVE, HYPNOTIC OR ANXIOLYTIC ABUSE, EPISODIC
305.7	AMPHETAMINE OR RELATED ACTING SYMPATHOMIMETIC ABUSE UNSPECIFIED
305.91	OTHER, MIXED, OR UNSPECIFIED DRUG ABSE, CONTINUOUS USE
304	OPIOID TYPE DEPENDENCE UNSPECIFIED
304.01	OPIOID TYPE DEPENDENCE CONTINUOUS
304.12	BARBITURATE AND SIMILARLY ACTING SEDATIVE OR HYPNOTIC DEPENDENCE EPISODIC
304.12	SEDATIVE, HYPNOTIC OR ANXIOLYTIC DEPENDENCE, EPISODIC
304.21	COCAINE DEPENDENCE, CONTINUOUS USE
304.4	AMPHETAMINE AND OTHER PSYCHOSTIMULANT DEPENDENCE UNSPECIFIED
304.6	OTHER SPECIFIED DRUG DEPENDENCE, UNSPECIFIED USE
304.91	UNSPECIFIED DRUG DEPENDENCE, CONTINUOUS
304.92	UNSPECIFIED DRUG DEPENDENCE, EPISODIC USE
305.02	ALCOHOL ABUSE, EPISODIC DRINKING BEHAVIOR
305.21	CANNABIS ABUSE, CONTINUOUS USE
305.52	OPIOID ABUSE EPISODIC
305.61	COCAINE ABUSE, CONTINUOUS USE
305.72	AMPHETAMINE OR RELATED ACTING SYMPATHOMIMETIC ABUSE EPISODIC
305.9	OTHER, MIXED, OR UNSPECIFIED DRUG ABUSE, UNSPECIFIED USE
305.92	OTHER MIXED, OR UNSPECIFIED DRUG ABUSE, EPISODIC USE
303.92	OTHER AND UNSPECIFIED ALCOHOL DEPENDENCE, EPISODIC DRINKING BEHAVIOR
304.02	OPIOID TYPE DEPENDENCE EPISODIC
304.11	BARBITURATE AND SIMILARLY ACTING SEDATIVE OR HYPNOTIC DEPENDENCE CONTINUOUS
304.2	COCAINE DEPENDENCE, UNSPECIFIED USE
304.3	CANNABIS DEPENDENCE UNSPECIFIED
304.5	HALLUCINOGEN DEPENDENCE UNSPECIFIED
304.52	HALLUCINOGEN DEPENDENCE, EPISODIC USE
304.62	OTHER SPECIFIED DRUG DEPENDENCE, EPISODIC USE
304.7	COMBINATIONS OF OPIOID TYPE DRUG WITH ANY OTHER DRUG DEPENDENCE, UNSPECIFIED USE
304.72	COMBINATIONS OF OPIOID TYPE DRUG WITH ANY OTHER DRUG DEPENDENCE, EPISODIC USE
304.81	COMBINATIONS OF DRUG DEPENDENCE EXCLUDING OPIOID TYPE DRUG, CONTINUOUS USE
304.82	COMBINATIONS OF DRUG DEPENDENCE EXCLUDING OPIOID TYPE DRUG, EPISODIC USE
305	ALCOHOL ABUSE, UNSPECIFIED DRINKING BEHAVIOR
305.31	HALLUCINOGEN ABUSE, CONTINUOUS USE
305.4	SEDATIVE, HYPNOTIC OR ANXIOLYTIC ABUSE, UNSPECIFIED
305.41	BARBITURATE AND SIMILARLY ACTING SEDATIVE OR HYPNOTIC ABUSE CONTINUOUS
305.62	COCAINE ABUSE, EPISODIC USE
303.91	OTHER AND UNSPECIFIED ALCOHOL DEPENDENCE, CONTINUOUS DRINKING BEHAVIOR
304.1	BARBITURATE AND SIMILARLY ACTING SEDATIVE OR HYPNOTIC DEPENDENCE UNSPECIFIED

© 2020 Leung LB et al. *JAMA Network Open.*

304.11	SEDATIVE, HYPNOTIC OR ANXIOLYTIC DEPENDENCE, CONTINUOUS
304.31	CANNABIS DEPENDENCE, CONTINUOUS USE
304.32	CANNABIS DEPENDENCE, EPISODIC USE
304.41	AMPHETAMINE AND OTHER PSYCHOSTIMULANT DEPENDENCE CONTINUOUS
304.51	HALLUCINOGEN DEPENDENCE, CONTINUOUS USE
304.71	COMBINATIONS OF OPIOID TYPE DRUG WITH ANY OTHER DRUG DEPENDENCE, CONTINUOUS USE
304.8	COMBINATIONS OF DRUG DEPENDENCE EXCLUDING OPIOID TYPE DRUG, UNSPECIFIED USE
304.9	UNSPECIFIED DRUG DEPENDENCE NEC, UNSPECIFIED
305.01	ALCOHOL ABUSE, CONTINUOUS DRINKING BEHAVIOR
305.2	CANNABIS ABUSE UNSPECIFIED
305.42	BARBITURATE AND SIMILARLY ACTING SEDATIVE OR HYPNOTIC ABUSE EPISODIC
305.5	OPIOID ABUSE UNSPECIFIED
305.51	OPIOID ABUSE CONTINUOUS
305.6	COCAINE ABUSE UNSPECIFIED
305.71	AMPHETAMINE OR RELATED ACTING SYMPATHOMIMETIC ABUSE CONTINUOUS
ANXIETY	
300	ANXIETY STATE, UNSPECIFIED
300.01	PANIC DISORDER
300.01	PANIC DISORDER WITHOUT AGORAPHOBIA
300.02	GENERALIZED ANXIETY DISORDER
300.09	OTHER ANXIETY STATES
300.2	PHOBIA, UNSPECIFIED
300.21	AGORAPHOBIA WITH PANIC ATTACKS
300.21	AGORAPHOBIA WITH PANIC DISORDER
300.22	AGORAPHOBIA WITHOUT MENTION OF PANIC ATTACKS
300.23	SOCIAL PHOBIA
SMI	
295	SIMPLE TYPE SCHIZOPHRENIA, UNSPECIFIED STATE
295.01	SIMPLE TYPE SCHIZOPHRENIA, SUBCHRONIC STATE
295.02	SIMPLE TYPE SCHIZOPHRENIA, CHRONIC STATE
295.03	SIMPLE TYPE SCHIZOPHRENIA, SUBCHRONIC STATE WITH ACUTE EXACERBATION
295.04	SIMPLE TYPE SCHIZOPHRENIA, CHRONIC STATE WITH ACUTE EXACERBATION
295.05	SIMPLE TYPE SCHIZOPHRENIA, IN REMISSION
295.1	DISORGANIZED TYPE SCHIZOPHRENIA, UNSPECIFIED STATE
295.11	DISORGANIZED TYPE SCHIZOPHRENIA, SUBCHRONIC STATE
295.12	DISORGANIZED TYPE SCHIZOPHRENIA, CHRONIC STATE
295.13	DISORGANIZED TYPE SCHIZOPHRENIA, SUBCHRONIC STATE WITH ACUTE EXACERBATION
295.14	DISORGANIZED TYPE SCHIZOPHRENIA, CHRONIC STATE WITH ACUTE EXACERBATION
295.15	DISORGANIZED TYPE SCHIZOPHRENIA, IN REMISSION
295.2	CATATONIC TYPE SCHIZOPHRENIA, UNSPECIFIED STATE
295.21	CATATONIC TYPE SCHIZOPHRENIA, SUBCHRONIC STATE

© 2020 Leung LB et al. *JAMA Network Open.*

295.22	CATATONIC TYPE SCHIZOPHRENIA, CHRONIC STATE
295.23	CATATONIC TYPE SCHIZOPHRENIA, SUBCHRONIC STATE WITH ACUTE EXACERBATION
295.24	CATATONIC TYPE SCHIZOPHRENIA, CHRONIC STATE WITH ACUTE EXACERBATION
295.25	CATATONIC TYPE SCHIZOPHRENIA, IN REMISSION
295.3	PARANOID TYPE SCHIZOPHRENIA, UNSPECIFIED STATE
295.31	PARANOID TYPE SCHIZOPHRENIA, SUBCHRONIC STATE
295.32	PARANOID TYPE SCHIZOPHRENIA, CHRONIC STATE
295.33	PARANOID TYPE SCHIZOPHRENIA, SUBCHRONIC STATE WITH ACUTE EXACERBATION
295.34	PARANOID TYPE SCHIZOPHRENIA, CHRONIC STATE WITH ACUTE EXACERBATION
295.35	PARANOID TYPE SCHIZOPHRENIA, IN REMISSION
295.4	ACUTE SCHIZOPHRENIC EPISODE, UNSPECIFIED STATE
295.4	SCHIZOPHRENIFORM DISORDER, UNSPECIFIED
295.41	ACUTE SCHIZOPHRENIC EPISODE, SUBCHRONIC STATE
295.41	SCHIZOPHRENIFORM DISORDER, SUBCHRONIC
295.42	ACUTE SCHIZOPHRENIC EPISODE, CHRONIC STATE
295.42	SCHIZOPHRENIFORM DISORDER, CHRONIC
295.43	ACUTE SCHIZOPHRENIC EPISODE, SUBCHRONIC STATE WITH ACUTE EXACERBATION
295.43	SCHIZOPHRENIFORM DISORDER, SUBCHRONIC WITH ACUTE EXACERBATION
295.44	ACUTE SCHIZOPHRENIC EPISODE, CHRONIC STATE WITH ACUTE EXACERBATION
295.44	SCHIZOPHRENIFORM DISORDER, CHRONIC WITH ACUTE EXACERBATION
295.45	ACUTE SCHIZOPHRENIC EPISODE, IN REMISSION
295.45	SCHIZOPHRENIFORM DISORDER, IN REMISSION
295.5	LATENT SCHIZOPHRENIA, UNSPECIFIED STATE
295.51	LATENT SCHIZOPHRENIA, SUBCHRONIC STATE
295.52	LATENT SCHIZOPHRENIA, CHRONIC STATE
295.53	LATENT SCHIZOPHRENIA, SUBCHRONIC STATE WITH ACUTE EXACERBATION
295.54	LATENT SCHIZOPHRENIA, CHRONIC STATE WITH ACUTE EXACERBATION
295.55	LATENT SCHIZOPHRENIA, IN REMISSION
295.6	RESIDUAL SCHIZOPHRENIA, UNSPECIFIED STATE
295.6	SCHIZOPHRENIC DISORDERS, RESIDUAL TYPE, UNSPECIFIED
295.61	RESIDUAL SCHIZOPHRENIA, SUBCHRONIC STATE
295.61	SCHIZOPHRENIC DISORDERS, RESIDUAL TYPE, SUBCHRONIC
295.62	RESIDUAL SCHIZOPHRENIA, CHRONIC STATE
295.62	SCHIZOPHRENIC DISORDERS, RESIDUAL TYPE, CHRONIC
295.63	RESIDUAL SCHIZOPHRENIA, SUBCHRONIC STATE WITH ACUTE EXACERBATION
295.63	SCHIZOPHRENIC DISORDERS, RESIDUAL TYPE, SUBCHRONIC WITH ACUTE EXACERBATION
295.64	RESIDUAL SCHIZOPHRENIA, CHRONIC STATE WITH ACUTE EXACERBATION
295.64	SCHIZOPHRENIC DISORDERS, RESIDUAL TYPE, CHRONIC WITH ACUTE EXACERBATION
295.65	RESIDUAL SCHIZOPHRENIA, IN REMISSION
295.65	SCHIZOPHRENIC DISORDERS, RESIDUAL TYPE, IN REMISSION
295.7	SCHIZO-AFFECTIVE TYPE SCHIZOPHRENIA, UNSPECIFIED STATE

© 2020 Leung LB et al. *JAMA Network Open.*

295.7	SCHIZOAFFECTIVE DISORDER, UNSPECIFIED
295.71	SCHIZO-AFFECTIVE TYPE SCHIZOPHRENIA, SUBCHRONIC STATE
295.71	SCHIZOAFFECTIVE DISORDER, SUBCHRONIC
295.72	SCHIZO-AFFECTIVE TYPE SCHIZOPHRENIA, CHRONIC STATE
295.72	SCHIZOAFFECTIVE DISORDER, CHRONIC
295.73	SCHIZO-AFFECTIVE TYPE SCHIZOPHRENIA, SUBCHRONIC STATE WITH ACUTE EXACERBATION
295.73	SCHIZOAFFECTIVE DISORDER, SUBCHRONIC WITH ACUTE EXACERBATION
295.74	SCHIZO-AFFECTIVE TYPE SCHIZOPHRENIA, CHRONIC STATE WITH ACUTE EXACERBATION
295.74	SCHIZOAFFECTIVE DISORDER, CHRONIC WITH ACUTE EXACERBATION
295.75	SCHIZO-AFFECTIVE TYPE SCHIZOPHRENIA, IN REMISSION
295.75	SCHIZOAFFECTIVE DISORDER, IN REMISSION
295.8	OTHER SPECIFIED TYPES OF SCHIZOPHRENIA, UNSPECIFIED STATE
295.81	OTHER SPECIFIED TYPES OF SCHIZOPHRENIA, SUBCHRONIC STATE
295.82	OTHER SPECIFIED TYPES OF SCHIZOPHRENIA, CHRONIC STATE
295.83	OTHER SPECIFIED TYPES OF SCHIZOPHRENIA, SUBCHRONIC STATE WITH ACUTE EXACERBATION
295.84	OTHER SPECIFIED TYPES OF SCHIZOPHRENIA, CHRONIC STATE WITH ACUTE EXACERBATION
295.85	OTHER SPECIFIED TYPES OF SCHIZOPHRENIA, IN REMISSION
295.9	UNSPECIFIED TYPE SCHIZOPHRENIA, UNSPECIFIED STATE
295.91	UNSPECIFIED TYPE SCHIZOPHRENIA, SUBCHRONIC STATE
295.92	UNSPECIFIED TYPE SCHIZOPHRENIA, CHRONIC STATE
295.93	UNSPECIFIED TYPE SCHIZOPHRENIA, SUBCHRONIC STATE WITH ACUTE EXACERBATION
295.94	UNSPECIFIED TYPE SCHIZOPHRENIA, CHRONIC STATE WITH ACUTE EXACERBATION
295.95	UNSPECIFIED TYPE SCHIZOPHRENIA, IN REMISSION
296	BIPOLAR I DISORDER, SINGLE MANIC EPISODE, UNSPECIFIED
296	MANIC AFFECTIVE DISORDER, SINGLE EPISODE, UNSPECIFIED DEGREE
296.01	BIPOLAR I DISORDER, SINGLE MANIC EPISODE, MILD
296.01	MANIC AFFECTIVE DISORDER, SINGLE EPISODE, MILD DEGREE
296.02	BIPOLAR I DISORDER, SINGLE MANIC EPISODE, MODERATE
296.02	MANIC AFFECTIVE DISORDER, SINGLE EPISODE, MODERATE DEGREE
296.03	BIPOLAR I DISORDER, SINGLE MANIC EPISODE, SEVERE, WITHOUT MENTION OF PSYCHOTIC BEHAVIOR
296.03	MANIC AFFECTIVE DISORDER, SINGLE EPISODE, SEVERE DEGREE, WITHOUT MENTION OF PSYCHOTIC BEHAVIOR
296.04	BIPOLAR I DISORDER, SINGLE MANIC EPISODE, SEVERE, SPECIFIED AS WITH PSYCHOTIC BEHAVIOR
296.04	MANIC AFFECTIVE DISORDER, SINGLE EPISODE, SEVERE DEGREE, SPECIFIED AS WITH PSYCHOTIC BEHAVIOR
296.05	BIPOLAR I DISORDER, SINGLE MANIC EPISODE, IN PARTIAL OR UNSPECIFIED REMISSION
296.05	MANIC AFFECTIVE DISORDER, SINGLE EPISODE, IN PARTIAL OR UNSPECIFIED REMISSION
296.06	BIPOLAR I DISORDER, SINGLE MANIC EPISODE, IN FULL REMISSION
296.06	MANIC AFFECTIVE DISORDER, SINGLE EPISODE, IN FULL REMISSION
296.1	MANIC AFFECTIVE DISORDER, RECURRENT EPISODE, UNSPECIFIED DEGREE
296.11	MANIC AFFECTIVE DISORDER, RECURRENT EPISODE, MILD DEGREE
296.12	MANIC AFFECTIVE DISORDER, RECURRENT EPISODE, MODERATE DEGREE

© 2020 Leung LB et al. *JAMA Network Open.*

296.13	MANIC AFFECTIVE DISORDER, RECURRENT EPISODE, SEVERE DEGREE, WITHOUT MENTION OF PSYCHOTIC BEHAVIOR
296.14	MANIC AFFECTIVE DISORDER, RECURRENT EPISODE, SEVERE DEGREE, SPECIFIED AS WITH PSYCHOTIC BEHAVIOR
296.15	MANIC AFFECTIVE DISORDER, RECURRENT EPISODE, IN PARTIAL OR UNSPECIFIED REMISSION
296.16	MANIC AFFECTIVE DISORDER, RECURRENT EPISODE, IN FULL REMISSION
296.4	BIPOLAR AFFECTIVE DISORDER, MANIC, UNSPECIFIED DEGREE
296.4	BIPOLAR I DISORDER, MOST RECENT EPISODE (OR CURRENT) MANIC, UNSPECIFIED
296.41	BIPOLAR AFFECTIVE DISORDER, MANIC, MILD DEGREE
296.41	BIPOLAR I DISORDER, MOST RECENT EPISODE (OR CURRENT) MANIC, MILD
296.42	BIPOLAR AFFECTIVE DISORDER, MANIC, MODERATE DEGREE
296.42	BIPOLAR I DISORDER, MOST RECENT EPISODE (OR CURRENT) MANIC, MODERATE
296.43	BIPOLAR AFFECTIVE DISORDER, MANIC, SEVERE DEGREE, WITHOUT MENTION OF PSYCHOTIC BEHAVIOR
296.43	BIPOLAR I DISORDER, MOST RECENT EPISODE (OR CURRENT) MANIC, SEVERE, WITHOUT MENTION OF PSYCHOTIC BEHAVIOR
296.44	BIPOLAR AFFECTIVE DISORDER, MANIC, SEVERE DEGREE, SPECIFIED AS WITH PSYCHOTIC BEHAVIOR
296.44	BIPOLAR I DISORDER, MOST RECENT EPISODE (OR CURRENT) MANIC, SEVERE, SPECIFIED AS WITH PSYCHOTIC BEHAVIOR
296.45	BIPOLAR AFFECTIVE DISORDER, MANIC, IN PARTIAL OR UNSPECIFIED REMISSION
296.45	BIPOLAR I DISORDER, MOST RECENT EPISODE (OR CURRENT) MANIC, IN PARTIAL OR UNSPECIFIED REMISSION
296.46	BIPOLAR AFFECTIVE DISORDER, MANIC, IN FULL REMISSION
296.46	BIPOLAR I DISORDER, MOST RECENT EPISODE (OR CURRENT) MANIC, IN FULL REMISSION
296.5	BIPOLAR AFFECTIVE DISORDER, DEPRESSED, UNSPECIFIED DEGREE
296.5	BIPOLAR I DISORDER, MOST RECENT EPISODE (OR CURRENT) DEPRESSED, UNSPECIFIED
296.51	BIPOLAR AFFECTIVE DISORDER, DEPRESSED, MILD DEGREE
296.51	BIPOLAR I DISORDER, MOST RECENT EPISODE (OR CURRENT) DEPRESSED, MILD
296.52	BIPOLAR AFFECTIVE DISORDER, DEPRESSED, MODERATE DEGREE
296.52	BIPOLAR I DISORDER, MOST RECENT EPISODE (OR CURRENT) DEPRESSED, MODERATE
296.53	BIPOLAR AFFECTIVE DISORDER, DEPRESSED, SEVERE DEGREE, WITHOUT MENTION OF PSYCHOTIC BEHAVIOR
296.53	BIPOLAR I DISORDER, MOST RECENT EPISODE (OR CURRENT) DEPRESSED, SEVERE, WITHOUT MENTION OF PSYCHOTIC BEHAVIOR
296.54	BIPOLAR AFFECTIVE DISORDER, DEPRESSED, SEVERE DEGREE, SPECIFIED AS WITH PSYCHOTIC BEHAVIOR
296.54	BIPOLAR I DISORDER, MOST RECENT EPISODE (OR CURRENT) DEPRESSED, SEVERE, SPECIFIED AS WITH PSYCHOTIC BEHAVIOR
296.55	BIPOLAR AFFECTIVE DISORDER, DEPRESSED, IN PARTIAL OR UNSPECIFIED REMISSION
296.55	BIPOLAR I DISORDER, MOST RECENT EPISODE (OR CURRENT) DEPRESSED, IN PARTIAL OR UNSPECIFIED REMISSION
296.56	BIPOLAR AFFECTIVE DISORDER, DEPRESSED, IN REMISSION
296.56	BIPOLAR I DISORDER, MOST RECENT EPISODE (OR CURRENT) DEPRESSED, IN FULL REMISSION
296.6	BIPOLAR AFFECTIVE DISORDER, MIXED, UNSPECIFIED DEGREE
296.6	BIPOLAR I DISORDER, MOST RECENT EPISODE (OR CURRENT) MIXED, UNSPECIFIED
296.61	BIPOLAR AFFECTIVE DISORDER, MIXED, MILD DEGREE
296.61	BIPOLAR I DISORDER, MOST RECENT EPISODE (OR CURRENT) MIXED, MILD

© 2020 Leung LB et al. *JAMA Network Open.*

296.62	BIPOLAR AFFECTIVE DISORDER, MIXED, MODERATE DEGREE
296.62	BIPOLAR I DISORDER, MOST RECENT EPISODE (OR CURRENT) MIXED, MODERATE
296.63	BIPOLAR AFFECTIVE DISORDER, MIXED, SEVERE DEGREE, WITHOUT MENTION OF PSYCHOTIC BEHAVIOR
296.63	BIPOLAR I DISORDER, MOST RECENT EPISODE (OR CURRENT) MIXED, SEVERE, WITHOUT MENTION OF PSYCHOTIC BEHAVIOR
296.64	BIPOLAR AFFECTIVE DISORDER, MIXED, SEVERE DEGREE, SPECIFIED AS WITH PSYCHOTIC BEHAVIOR
296.64	BIPOLAR I DISORDER, MOST RECENT EPISODE (OR CURRENT) MIXED, SEVERE, SPECIFIED AS WITH PSYCHOTIC BEHAVIOR
296.65	BIPOLAR AFFECTIVE DISORDER, MIXED, IN PARTIAL OR UNSPECIFIED REMISSION
296.65	BIPOLAR I DISORDER, MOST RECENT EPISODE (OR CURRENT) MIXED, IN PARTIAL OR UNSPECIFIED REMISSION
296.66	BIPOLAR AFFECTIVE DISORDER, MIXED, IN FULL REMISSION
296.66	BIPOLAR I DISORDER, MOST RECENT EPISODE (OR CURRENT) MIXED, IN FULL REMISSION
296.7	BIPOLAR AFFECTIVE DISORDER, UNSPECIFIED
296.7	BIPOLAR I DISORDER, MOST RECENT EPISODE (OR CURRENT) UNSPECIFIED
296.8	BIPOLAR DISORDER, UNSPECIFIED
296.8	MANIC-DEPRESSIVE PSYCHOSIS, UNSPECIFIED
296.81	ATYPICAL MANIC DISORDER
296.82	ATYPICAL DEPRESSIVE DISORDER
296.89	OTHER AND UNSPECIFIED BIPOLAR DISORDERS, OTHER
296.89	OTHER MANIC-DEPRESSIVE PSYCHOSIS

© 2020 Leung LB et al. *JAMA Network Open.*

ICD 10 Codes	
PTSD	
F43.10	POST-TRAUMATIC STRESS DISORDER, UNSPECIFIED
F43.11	POST-TRAUMATIC STRESS DISORDER, ACUTE
F43.12	POST-TRAUMATIC STRESS DISORDER, CHRONIC
ANXIETY	
F40.01	AGORAPHOBIA WITH PANIC DISORDER
F40.02	AGORAPHOBIA WITHOUT PANIC DISORDER
F40.11	SOCIAL PHOBIA, GENERALIZED
F40.241	ACROPHOBIA
F40.290	ANDROPHOBIA
F42.8	OTHER OBSESSIVE-COMPULSIVE DISORDER
F42.9	OBSESSIVE-COMPULSIVE DISORDER, UNSPECIFIED
F43.0	ACUTE STRESS REACTION
F44.5	CONVERSION DISORDER WITH SEIZURES OR CONVULSIONS
F44.9	DISSOCIATIVE AND CONVERSION DISORDER, UNSPECIFIED
F45.0	SOMATIZATION DISORDER
F45.9	SOMATOFORM DISORDER, UNSPECIFIED
F48.8	OTHER SPECIFIED NONPSYCHOTIC MENTAL DISORDERS
F48.9	NONPSYCHOTIC MENTAL DISORDER, UNSPECIFIED
F40.218	OTHER ANIMAL TYPE PHOBIA
F40.220	FEAR OF THUNDERSTORMS
F40.240	CLAUSTROPHOBIA
F40.298	OTHER SPECIFIED PHOBIA
F40.9	PHOBIC ANXIETY DISORDER, UNSPECIFIED
F41.0	PANIC DISORDER [EPISODIC PAROXYSMAL ANXIETY] WITHOUT AGORAPHOBIA
F41.1	GENERALIZED ANXIETY DISORDER
F41.3	OTHER MIXED ANXIETY DISORDERS
F41.8	OTHER SPECIFIED ANXIETY DISORDERS
F43.24	ADJUSTMENT DISORDER WITH DISTURBANCE OF CONDUCT
F43.9	REACTION TO SEVERE STRESS, UNSPECIFIED
F45.20	HYPOCHONDRIACAL DISORDER, UNSPECIFIED
F45.22	BODY DYSMORPHIC DISORDER
F45.29	OTHER HYPOCHONDRIACAL DISORDERS
F45.41	PAIN DISORDER EXCLUSIVELY RELATED TO PSYCHOLOGICAL FACTORS
F45.8	OTHER SOMATOFORM DISORDERS
F48.1	DEPERSONALIZATION-DEREALIZATION SYNDROME
F48.2	PSEUDOBULBAR AFFECT
F40.10	SOCIAL PHOBIA, UNSPECIFIED
F40.210	ARACHNOPHOBIA
F40.230	FEAR OF BLOOD

© 2020 Leung LB et al. *JAMA Network Open.*

F40.231	FEAR OF INJECTIONS AND TRANSFUSIONS
F40.232	FEAR OF OTHER MEDICAL CARE
F40.242	FEAR OF BRIDGES
F40.8	OTHER PHOBIC ANXIETY DISORDERS
F41.0	PANIC DISORDER [EPISODIC PAROXYSMAL ANXIETY]
F41.9	ANXIETY DISORDER, UNSPECIFIED
F42.	OBSESSIVE-COMPULSIVE DISORDER
F43.22	ADJUSTMENT DISORDER WITH ANXIETY
F43.8	OTHER REACTIONS TO SEVERE STRESS
F44.2	DISSOCIATIVE STUPOR
F44.89	OTHER DISSOCIATIVE AND CONVERSION DISORDERS
F45.1	UNDIFFERENTIATED SOMATOFORM DISORDER
F40.00	AGORAPHOBIA, UNSPECIFIED
F40.228	OTHER NATURAL ENVIRONMENT TYPE PHOBIA
F40.233	FEAR OF INJURY
F40.243	FEAR OF FLYING
F40.248	OTHER SITUATIONAL TYPE PHOBIA
F40.291	GYNEPHOBIA
F42.2	MIXED OBSESSIONAL THOUGHTS AND ACTS
F42.3	HOARDING DISORDER
F42.4	EXCORIATION (SKIN-PICKING) DISORDER
F43.20	ADJUSTMENT DISORDER, UNSPECIFIED
F43.21	ADJUSTMENT DISORDER WITH DEPRESSED MOOD
F43.23	ADJUSTMENT DISORDER WITH MIXED ANXIETY AND DEPRESSED MOOD
F43.25	ADJUSTMENT DISORDER WITH MIXED DISTURBANCE OF EMOTIONS AND CONDUCT
F43.29	ADJUSTMENT DISORDER WITH OTHER SYMPTOMS
F44.0	DISSOCIATIVE AMNESIA
F44.1	DISSOCIATIVE FUGUE
F44.4	CONVERSION DISORDER WITH MOTOR SYMPTOM OR DEFICIT
F44.6	CONVERSION DISORDER WITH SENSORY SYMPTOM OR DEFICIT
F44.7	CONVERSION DISORDER WITH MIXED SYMPTOM PRESENTATION
F44.81	DISSOCIATIVE IDENTITY DISORDER
F45.21	HYPOCHONDRIASIS
F45.42	PAIN DISORDER WITH RELATED PSYCHOLOGICAL FACTORS
SCHIZ	
F20.2	CATATONIC SCHIZOPHRENIA
F20.0	PARANOID SCHIZOPHRENIA
F20.1	DISORGANIZED SCHIZOPHRENIA
F20.89	OTHER SCHIZOPHRENIA
F20.3	UNDIFFERENTIATED SCHIZOPHRENIA
F20.5	RESIDUAL SCHIZOPHRENIA

© 2020 Leung LB et al. *JAMA Network Open.*

F20.81	SCHIZOPHRENIFORM DISORDER
F20.9	SCHIZOPHRENIA, UNSPECIFIED
BIPOLAR	
F31.4	BIPOLAR DISORDER, CURRENT EPISODE DEPRESSED, SEVERE, WITHOUT PSYCHOTIC FEATURES
F31.74	BIPOLAR DISORDER, IN FULL REMISSION, MOST RECENT EPISODE MANIC
F31.75	BIPOLAR DISORDER, IN PARTIAL REMISSION, MOST RECENT EPISODE DEPRESSED
F31.78	BIPOLAR DISORDER, IN FULL REMISSION, MOST RECENT EPISODE MIXED
F31.89	OTHER BIPOLAR DISORDER
F31.9	BIPOLAR DISORDER, UNSPECIFIED
F31.10	BIPOLAR DISORDER, CURRENT EPISODE MANIC WITHOUT PSYCHOTIC FEATURES, UNSPECIFIED
F31.11	BIPOLAR DISORDER, CURRENT EPISODE MANIC WITHOUT PSYCHOTIC FEATURES, MILD
F31.31	BIPOLAR DISORDER, CURRENT EPISODE DEPRESSED, MILD
F31.60	BIPOLAR DISORDER, CURRENT EPISODE MIXED, UNSPECIFIED
F31.70	BIPOLAR DISORDER, CURRENTLY IN REMISSION, MOST RECENT EPISODE UNSPECIFIED
F31.77	BIPOLAR DISORDER, IN PARTIAL REMISSION, MOST RECENT EPISODE MIXED
F31.13	BIPOLAR DISORDER, CURRENT EPISODE MANIC WITHOUT PSYCHOTIC FEATURES, SEVERE
F31.2	BIPOLAR DISORDER, CURRENT EPISODE MANIC SEVERE WITH PSYCHOTIC FEATURES
F31.32	BIPOLAR DISORDER, CURRENT EPISODE DEPRESSED, MODERATE
F31.5	BIPOLAR DISORDER, CURRENT EPISODE DEPRESSED, SEVERE, WITH PSYCHOTIC FEATURES
F31.63	BIPOLAR DISORDER, CURRENT EPISODE MIXED, SEVERE, WITHOUT PSYCHOTIC FEATURES
F31.71	BIPOLAR DISORDER, IN PARTIAL REMISSION, MOST RECENT EPISODE HYPOMANIC
F31.76	BIPOLAR DISORDER, IN FULL REMISSION, MOST RECENT EPISODE DEPRESSED
F31.0	BIPOLAR DISORDER, CURRENT EPISODE HYPOMANIC
F31.12	BIPOLAR DISORDER, CURRENT EPISODE MANIC WITHOUT PSYCHOTIC FEATURES, MODERATE
F31.30	BIPOLAR DISORDER, CURRENT EPISODE DEPRESSED, MILD OR MODERATE SEVERITY, UNSPECIFIED
F31.61	BIPOLAR DISORDER, CURRENT EPISODE MIXED, MILD
F31.62	BIPOLAR DISORDER, CURRENT EPISODE MIXED, MODERATE
F31.64	BIPOLAR DISORDER, CURRENT EPISODE MIXED, SEVERE, WITH PSYCHOTIC FEATURES
F31.72	BIPOLAR DISORDER, IN FULL REMISSION, MOST RECENT EPISODE HYPOMANIC
F31.73	BIPOLAR DISORDER, IN PARTIAL REMISSION, MOST RECENT EPISODE MANIC
F31.81	BIPOLAR II DISORDER
DEPRESSION	
F32.0	MAJOR DEPRESSIVE DISORDER, SINGLE EPISODE, MILD
F33.0	MAJOR DEPRESSIVE DISORDER, RECURRENT, MILD
F33.3	MAJOR DEPRESSIVE DISORDER, RECURRENT, SEVERE WITH PSYCHOTIC SYMPTOMS
F32.2	MAJOR DEPRESSIVE DISORDER, SINGLE EPISODE, SEVERE WITHOUT PSYCHOTIC FEATURES
F32.4	MAJOR DEPRESSIVE DISORDER, SINGLE EPISODE, IN PARTIAL REMISSION
F33.2	MAJOR DEPRESSIVE DISORDER, RECURRENT SEVERE WITHOUT PSYCHOTIC FEATURES
F33.8	OTHER RECURRENT DEPRESSIVE DISORDERS
F32.1	MAJOR DEPRESSIVE DISORDER, SINGLE EPISODE, MODERATE
F32.5	MAJOR DEPRESSIVE DISORDER, SINGLE EPISODE, IN FULL REMISSION

© 2020 Leung LB et al. *JAMA Network Open.*

F32.9	MAJOR DEPRESSIVE DISORDER, SINGLE EPISODE, UNSPECIFIED
F33.1	MAJOR DEPRESSIVE DISORDER, RECURRENT, MODERATE
F33.40	MAJOR DEPRESSIVE DISORDER, RECURRENT, IN REMISSION, UNSPECIFIED
F32.3	MAJOR DEPRESSIVE DISORDER, SINGLE EPISODE, SEVERE WITH PSYCHOTIC FEATURES
F32.8	OTHER DEPRESSIVE EPISODES
F32.81	PREMENSTRUAL DYSPHORIC DISORDER
F32.89	OTHER SPECIFIED DEPRESSIVE EPISODES
F33.41	MAJOR DEPRESSIVE DISORDER, RECURRENT, IN PARTIAL REMISSION
F33.42	MAJOR DEPRESSIVE DISORDER, RECURRENT, IN FULL REMISSION
F33.9	MAJOR DEPRESSIVE DISORDER, RECURRENT, UNSPECIFIED
SMMENTAL	
F20.2	CATATONIC SCHIZOPHRENIA
F31.4	BIPOLAR DISORDER, CURRENT EPISODE DEPRESSED, SEVERE, WITHOUT PSYCHOTIC FEATURES
F31.74	BIPOLAR DISORDER, IN FULL REMISSION, MOST RECENT EPISODE MANIC
F31.75	BIPOLAR DISORDER, IN PARTIAL REMISSION, MOST RECENT EPISODE DEPRESSED
F31.78	BIPOLAR DISORDER, IN FULL REMISSION, MOST RECENT EPISODE MIXED
F31.89	OTHER BIPOLAR DISORDER
F31.9	BIPOLAR DISORDER, UNSPECIFIED
F20.3	UNDIFFERENTIATED SCHIZOPHRENIA
F20.5	RESIDUAL SCHIZOPHRENIA
F20.81	SCHIZOPHRENIFORM DISORDER
F20.9	SCHIZOPHRENIA, UNSPECIFIED
F31.10	BIPOLAR DISORDER, CURRENT EPISODE MANIC WITHOUT PSYCHOTIC FEATURES, UNSPECIFIED
F31.11	BIPOLAR DISORDER, CURRENT EPISODE MANIC WITHOUT PSYCHOTIC FEATURES, MILD
F31.31	BIPOLAR DISORDER, CURRENT EPISODE DEPRESSED, MILD
F31.60	BIPOLAR DISORDER, CURRENT EPISODE MIXED, UNSPECIFIED
F31.70	BIPOLAR DISORDER, CURRENTLY IN REMISSION, MOST RECENT EPISODE UNSPECIFIED
F31.77	BIPOLAR DISORDER, IN PARTIAL REMISSION, MOST RECENT EPISODE MIXED
F20.0	PARANOID SCHIZOPHRENIA
F31.13	BIPOLAR DISORDER, CURRENT EPISODE MANIC WITHOUT PSYCHOTIC FEATURES, SEVERE
F31.2	BIPOLAR DISORDER, CURRENT EPISODE MANIC SEVERE WITH PSYCHOTIC FEATURES
F31.32	BIPOLAR DISORDER, CURRENT EPISODE DEPRESSED, MODERATE
F31.5	BIPOLAR DISORDER, CURRENT EPISODE DEPRESSED, SEVERE, WITH PSYCHOTIC FEATURES
F31.63	BIPOLAR DISORDER, CURRENT EPISODE MIXED, SEVERE, WITHOUT PSYCHOTIC FEATURES
F31.71	BIPOLAR DISORDER, IN PARTIAL REMISSION, MOST RECENT EPISODE HYPOMANIC
F31.76	BIPOLAR DISORDER, IN FULL REMISSION, MOST RECENT EPISODE DEPRESSED
F20.1	DISORGANIZED SCHIZOPHRENIA
F20.89	OTHER SCHIZOPHRENIA
F21.	SCHIZOTYPAL DISORDER
F25.0	SCHIZOAFFECTIVE DISORDER, BIPOLAR TYPE
F25.1	SCHIZOAFFECTIVE DISORDER, DEPRESSIVE TYPE

© 2020 Leung LB et al. *JAMA Network Open.*

F25.8	OTHER SCHIZOAFFECTIVE DISORDERS
F25.9	SCHIZOAFFECTIVE DISORDER, UNSPECIFIED
F31.0	BIPOLAR DISORDER, CURRENT EPISODE HYPOMANIC
F31.12	BIPOLAR DISORDER, CURRENT EPISODE MANIC WITHOUT PSYCHOTIC FEATURES, MODERATE
F31.30	BIPOLAR DISORDER, CURRENT EPISODE DEPRESSED, MILD OR MODERATE SEVERITY, UNSPECIFIED
F31.61	BIPOLAR DISORDER, CURRENT EPISODE MIXED, MILD
F31.62	BIPOLAR DISORDER, CURRENT EPISODE MIXED, MODERATE
F31.64	BIPOLAR DISORDER, CURRENT EPISODE MIXED, SEVERE, WITH PSYCHOTIC FEATURES
F31.72	BIPOLAR DISORDER, IN FULL REMISSION, MOST RECENT EPISODE HYPOMANIC
F31.73	BIPOLAR DISORDER, IN PARTIAL REMISSION, MOST RECENT EPISODE MANIC
F31.81	BIPOLAR II DISORDER
OTHPSYCHO	
F22.	DELUSIONAL DISORDERS
F28.	OTHER PSYCHOTIC DISORDER NOT DUE TO A SUBSTANCE OR KNOWN PHYSIOLOGICAL CONDITION
F23.	BRIEF PSYCHOTIC DISORDER
F24.	SHARED PSYCHOTIC DISORDER
F29.	UNSPECIFIED PSYCHOSIS NOT DUE TO A SUBSTANCE OR KNOWN PHYSIOLOGICAL CONDITION
SABUSE	
F10.150	ALCOHOL ABUSE WITH ALCOHOL-INDUCED PSYCHOTIC DISORDER WITH DELUSIONS
F10.151	ALCOHOL ABUSE WITH ALCOHOL-INDUCED PSYCHOTIC DISORDER WITH HALLUCINATIONS
F10.182	ALCOHOL ABUSE WITH ALCOHOL-INDUCED SLEEP DISORDER
F10.188	ALCOHOL ABUSE WITH OTHER ALCOHOL-INDUCED DISORDER
F10.19	ALCOHOL ABUSE WITH UNSPECIFIED ALCOHOL-INDUCED DISORDER
F10.21	ALCOHOL DEPENDENCE, IN REMISSION
F10.221	ALCOHOL DEPENDENCE WITH INTOXICATION DELIRIUM
F10.229	ALCOHOL DEPENDENCE WITH INTOXICATION, UNSPECIFIED
F10.250	ALCOHOL DEPENDENCE WITH ALCOHOL-INDUCED PSYCHOTIC DISORDER WITH DELUSIONS
F10.259	ALCOHOL DEPENDENCE WITH ALCOHOL-INDUCED PSYCHOTIC DISORDER, UNSPECIFIED
F10.980	ALCOHOL USE, UNSPECIFIED WITH ALCOHOL-INDUCED ANXIETY DISORDER
F11.120	OPIOID ABUSE WITH INTOXICATION, UNCOMPLICATED
F11.122	OPIOID ABUSE WITH INTOXICATION WITH PERCEPTUAL DISTURBANCE
F11.282	OPIOID DEPENDENCE WITH OPIOID-INDUCED SLEEP DISORDER
F11.920	OPIOID USE, UNSPECIFIED WITH INTOXICATION, UNCOMPLICATED
F11.921	OPIOID USE, UNSPECIFIED WITH INTOXICATION DELIRIUM
F11.929	OPIOID USE, UNSPECIFIED WITH INTOXICATION, UNSPECIFIED
F11.93	OPIOID USE, UNSPECIFIED WITH WITHDRAWAL
F11.988	OPIOID USE, UNSPECIFIED WITH OTHER OPIOID-INDUCED DISORDER
F12.11	CANNABIS ABUSE, IN REMISSION
F12.121	CANNABIS ABUSE WITH INTOXICATION DELIRIUM
F12.150	CANNABIS ABUSE WITH PSYCHOTIC DISORDER WITH DELUSIONS
F12.159	CANNABIS ABUSE WITH PSYCHOTIC DISORDER, UNSPECIFIED

© 2020 Leung LB et al. *JAMA Network Open.*

F12.23	CANNABIS DEPENDENCE WITH WITHDRAWAL
F12.251	CANNABIS DEPENDENCE WITH PSYCHOTIC DISORDER WITH HALLUCINATIONS
F12.280	CANNABIS DEPENDENCE WITH CANNABIS-INDUCED ANXIETY DISORDER
F12.90	CANNABIS USE, UNSPECIFIED, UNCOMPLICATED
F12.929	CANNABIS USE, UNSPECIFIED WITH INTOXICATION, UNSPECIFIED
F12.980	CANNABIS USE, UNSPECIFIED WITH ANXIETY DISORDER
F12.99	CANNABIS USE, UNSPECIFIED WITH UNSPECIFIED CANNABIS-INDUCED DISORDER
F13.10	SEDATIVE, HYPNOTIC OR ANXIOLYTIC ABUSE, UNCOMPLICATED
F13.11	SEDATIVE, HYPNOTIC OR ANXIOLYTIC ABUSE, IN REMISSION
F13.129	SEDATIVE, HYPNOTIC OR ANXIOLYTIC ABUSE WITH INTOXICATION, UNSPECIFIED
F13.150	SEDATIVE, HYPNOTIC OR ANXIOLYTIC ABUSE WITH SEDATIVE, HYPNOTIC OR ANXIOLYTIC-INDUCED PSYCHOTIC DISORDER WITH DELUSIONS
F13.221	SEDATIVE, HYPNOTIC OR ANXIOLYTIC DEPENDENCE WITH INTOXICATION DELIRIUM
F13.229	SEDATIVE, HYPNOTIC OR ANXIOLYTIC DEPENDENCE WITH INTOXICATION, UNSPECIFIED
F13.230	SEDATIVE, HYPNOTIC OR ANXIOLYTIC DEPENDENCE WITH WITHDRAWAL, UNCOMPLICATED
F13.232	SEDATIVE, HYPNOTIC OR ANXIOLYTIC DEPENDENCE WITH WITHDRAWAL WITH PERCEPTUAL DISTURBANCE
F13.239	SEDATIVE, HYPNOTIC OR ANXIOLYTIC DEPENDENCE WITH WITHDRAWAL, UNSPECIFIED
F13.27	SEDATIVE, HYPNOTIC OR ANXIOLYTIC DEPENDENCE WITH SEDATIVE, HYPNOTIC OR ANXIOLYTIC-INDUCED PERSISTING DEMENTIA
F13.90	SEDATIVE, HYPNOTIC, OR ANXIOLYTIC USE, UNSPECIFIED, UNCOMPLICATED
F13.950	SEDATIVE, HYPNOTIC OR ANXIOLYTIC USE, UNSPECIFIED WITH SEDATIVE, HYPNOTIC OR ANXIOLYTIC-INDUCED PSYCHOTIC DISORDER WITH DELUSIONS
F13.981	SEDATIVE, HYPNOTIC OR ANXIOLYTIC USE, UNSPECIFIED WITH SEDATIVE, HYPNOTIC OR ANXIOLYTIC-INDUCED SEXUAL DYSFUNCTION
F13.982	SEDATIVE, HYPNOTIC OR ANXIOLYTIC USE, UNSPECIFIED WITH SEDATIVE, HYPNOTIC OR ANXIOLYTIC-INDUCED SLEEP DISORDER
F14.122	COCAINE ABUSE WITH INTOXICATION WITH PERCEPTUAL DISTURBANCE
F14.129	COCAINE ABUSE WITH INTOXICATION, UNSPECIFIED
F14.181	COCAINE ABUSE WITH COCAINE-INDUCED SEXUAL DYSFUNCTION
F14.182	COCAINE ABUSE WITH COCAINE-INDUCED SLEEP DISORDER
F14.229	COCAINE DEPENDENCE WITH INTOXICATION, UNSPECIFIED
F14.24	COCAINE DEPENDENCE WITH COCAINE-INDUCED MOOD DISORDER
F14.250	COCAINE DEPENDENCE WITH COCAINE-INDUCED PSYCHOTIC DISORDER WITH DELUSIONS
F14.921	COCAINE USE, UNSPECIFIED WITH INTOXICATION DELIRIUM
F10.120	ALCOHOL ABUSE WITH INTOXICATION, UNCOMPLICATED
F10.121	ALCOHOL ABUSE WITH INTOXICATION DELIRIUM
F10.14	ALCOHOL ABUSE WITH ALCOHOL-INDUCED MOOD DISORDER
F10.181	ALCOHOL ABUSE WITH ALCOHOL-INDUCED SEXUAL DYSFUNCTION
F10.220	ALCOHOL DEPENDENCE WITH INTOXICATION, UNCOMPLICATED
F10.27	ALCOHOL DEPENDENCE WITH ALCOHOL-INDUCED PERSISTING DEMENTIA
F10.280	ALCOHOL DEPENDENCE WITH ALCOHOL-INDUCED ANXIETY DISORDER
F10.950	ALCOHOL USE, UNSPECIFIED WITH ALCOHOL-INDUCED PSYCHOTIC DISORDER WITH DELUSIONS
F10.97	ALCOHOL USE, UNSPECIFIED WITH ALCOHOL-INDUCED PERSISTING DEMENTIA

© 2020 Leung LB et al. *JAMA Network Open.*

F10.981	ALCOHOL USE, UNSPECIFIED WITH ALCOHOL-INDUCED SEXUAL DYSFUNCTION
F10.982	ALCOHOL USE, UNSPECIFIED WITH ALCOHOL-INDUCED SLEEP DISORDER
F10.988	ALCOHOL USE, UNSPECIFIED WITH OTHER ALCOHOL-INDUCED DISORDER
F10.99	ALCOHOL USE, UNSPECIFIED WITH UNSPECIFIED ALCOHOL-INDUCED DISORDER
F11.10	OPIOID ABUSE, UNCOMPLICATED
F11.11	OPIOID ABUSE, IN REMISSION
F11.129	OPIOID ABUSE WITH INTOXICATION, UNSPECIFIED
F11.181	OPIOID ABUSE WITH OPIOID-INDUCED SEXUAL DYSFUNCTION
F11.20	OPIOID DEPENDENCE, UNCOMPLICATED
F11.21	OPIOID DEPENDENCE, IN REMISSION
F11.221	OPIOID DEPENDENCE WITH INTOXICATION DELIRIUM
F11.222	OPIOID DEPENDENCE WITH INTOXICATION WITH PERCEPTUAL DISTURBANCE
F11.229	OPIOID DEPENDENCE WITH INTOXICATION, UNSPECIFIED
F11.251	OPIOID DEPENDENCE WITH OPIOID-INDUCED PSYCHOTIC DISORDER WITH HALLUCINATIONS
F11.259	OPIOID DEPENDENCE WITH OPIOID-INDUCED PSYCHOTIC DISORDER, UNSPECIFIED
F11.90	OPIOID USE, UNSPECIFIED, UNCOMPLICATED
F11.94	OPIOID USE, UNSPECIFIED WITH OPIOID-INDUCED MOOD DISORDER
F11.981	OPIOID USE, UNSPECIFIED WITH OPIOID-INDUCED SEXUAL DYSFUNCTION
F11.982	OPIOID USE, UNSPECIFIED WITH OPIOID-INDUCED SLEEP DISORDER
F12.10	CANNABIS ABUSE, UNCOMPLICATED
F12.180	CANNABIS ABUSE WITH CANNABIS-INDUCED ANXIETY DISORDER
F12.188	CANNABIS ABUSE WITH OTHER CANNABIS-INDUCED DISORDER
F12.250	CANNABIS DEPENDENCE WITH PSYCHOTIC DISORDER WITH DELUSIONS
F12.950	CANNABIS USE, UNSPECIFIED WITH PSYCHOTIC DISORDER WITH DELUSIONS
F13.121	SEDATIVE, HYPNOTIC OR ANXIOLYTIC ABUSE WITH INTOXICATION DELIRIUM
F13.151	SEDATIVE, HYPNOTIC OR ANXIOLYTIC ABUSE WITH SEDATIVE, HYPNOTIC OR ANXIOLYTIC-INDUCED PSYCHOTIC DISORDER WITH HALLUCINATIONS
F13.181	SEDATIVE, HYPNOTIC OR ANXIOLYTIC ABUSE WITH SEDATIVE, HYPNOTIC OR ANXIOLYTIC-INDUCED SEXUAL DYSFUNCTION
F13.182	SEDATIVE, HYPNOTIC OR ANXIOLYTIC ABUSE WITH SEDATIVE, HYPNOTIC OR ANXIOLYTIC-INDUCED SLEEP DISORDER
F13.188	SEDATIVE, HYPNOTIC OR ANXIOLYTIC ABUSE WITH OTHER SEDATIVE, HYPNOTIC OR ANXIOLYTIC-INDUCED DISORDER
F13.20	SEDATIVE, HYPNOTIC OR ANXIOLYTIC DEPENDENCE, UNCOMPLICATED
F13.21	SEDATIVE, HYPNOTIC OR ANXIOLYTIC DEPENDENCE, IN REMISSION
F13.231	SEDATIVE, HYPNOTIC OR ANXIOLYTIC DEPENDENCE WITH WITHDRAWAL DELIRIUM
F13.24	SEDATIVE, HYPNOTIC OR ANXIOLYTIC DEPENDENCE WITH SEDATIVE, HYPNOTIC OR ANXIOLYTIC-INDUCED MOOD DISORDER
F13.251	SEDATIVE, HYPNOTIC OR ANXIOLYTIC DEPENDENCE WITH SEDATIVE, HYPNOTIC OR ANXIOLYTIC-INDUCED PSYCHOTIC DISORDER WITH HALLUCINATIONS
F13.280	SEDATIVE, HYPNOTIC OR ANXIOLYTIC DEPENDENCE WITH SEDATIVE, HYPNOTIC OR ANXIOLYTIC-INDUCED ANXIETY DISORDER
F13.281	SEDATIVE, HYPNOTIC OR ANXIOLYTIC DEPENDENCE WITH SEDATIVE, HYPNOTIC OR ANXIOLYTIC-INDUCED SEXUAL DYSFUNCTION

© 2020 Leung LB et al. *JAMA Network Open.*

F13.921	SEDATIVE, HYPNOTIC OR ANXIOLYTIC USE, UNSPECIFIED WITH INTOXICATION DELIRIUM
F13.929	SEDATIVE, HYPNOTIC OR ANXIOLYTIC USE, UNSPECIFIED WITH INTOXICATION, UNSPECIFIED
F13.94	SEDATIVE, HYPNOTIC OR ANXIOLYTIC USE, UNSPECIFIED WITH SEDATIVE, HYPNOTIC OR ANXIOLYTIC-INDUCED MOOD DISORDER
F13.959	SEDATIVE, HYPNOTIC OR ANXIOLYTIC USE, UNSPECIFIED WITH SEDATIVE, HYPNOTIC OR ANXIOLYTIC-INDUCED PSYCHOTIC DISORDER, UNSPECIFIED
F13.96	SEDATIVE, HYPNOTIC OR ANXIOLYTIC USE, UNSPECIFIED WITH SEDATIVE, HYPNOTIC OR ANXIOLYTIC-INDUCED PERSISTING AMNESTIC DISORDER
F13.97	SEDATIVE, HYPNOTIC OR ANXIOLYTIC USE, UNSPECIFIED WITH SEDATIVE, HYPNOTIC OR ANXIOLYTIC-INDUCED PERSISTING DEMENTIA
F14.188	COCAINE ABUSE WITH OTHER COCAINE-INDUCED DISORDER
F14.19	COCAINE ABUSE WITH UNSPECIFIED COCAINE-INDUCED DISORDER
F14.220	COCAINE DEPENDENCE WITH INTOXICATION, UNCOMPLICATED
F14.282	COCAINE DEPENDENCE WITH COCAINE-INDUCED SLEEP DISORDER
F14.920	COCAINE USE, UNSPECIFIED WITH INTOXICATION, UNCOMPLICATED
F10.11	ALCOHOL ABUSE, IN REMISSION
F10.129	ALCOHOL ABUSE WITH INTOXICATION, UNSPECIFIED
F10.159	ALCOHOL ABUSE WITH ALCOHOL-INDUCED PSYCHOTIC DISORDER, UNSPECIFIED
F10.20	ALCOHOL DEPENDENCE, UNCOMPLICATED
F10.231	ALCOHOL DEPENDENCE WITH WITHDRAWAL DELIRIUM
F10.232	ALCOHOL DEPENDENCE WITH WITHDRAWAL WITH PERCEPTUAL DISTURBANCE
F10.239	ALCOHOL DEPENDENCE WITH WITHDRAWAL, UNSPECIFIED
F10.24	ALCOHOL DEPENDENCE WITH ALCOHOL-INDUCED MOOD DISORDER
F10.26	ALCOHOL DEPENDENCE WITH ALCOHOL-INDUCED PERSISTING AMNESTIC DISORDER
F10.281	ALCOHOL DEPENDENCE WITH ALCOHOL-INDUCED SEXUAL DYSFUNCTION
F10.282	ALCOHOL DEPENDENCE WITH ALCOHOL-INDUCED SLEEP DISORDER
F10.288	ALCOHOL DEPENDENCE WITH OTHER ALCOHOL-INDUCED DISORDER
F10.29	ALCOHOL DEPENDENCE WITH UNSPECIFIED ALCOHOL-INDUCED DISORDER
F10.94	ALCOHOL USE, UNSPECIFIED WITH ALCOHOL-INDUCED MOOD DISORDER
F11.121	OPIOID ABUSE WITH INTOXICATION DELIRIUM
F11.14	OPIOID ABUSE WITH OPIOID-INDUCED MOOD DISORDER
F11.150	OPIOID ABUSE WITH OPIOID-INDUCED PSYCHOTIC DISORDER WITH DELUSIONS
F11.151	OPIOID ABUSE WITH OPIOID-INDUCED PSYCHOTIC DISORDER WITH HALLUCINATIONS
F11.159	OPIOID ABUSE WITH OPIOID-INDUCED PSYCHOTIC DISORDER, UNSPECIFIED
F11.23	OPIOID DEPENDENCE WITH WITHDRAWAL
F11.250	OPIOID DEPENDENCE WITH OPIOID-INDUCED PSYCHOTIC DISORDER WITH DELUSIONS
F11.29	OPIOID DEPENDENCE WITH UNSPECIFIED OPIOID-INDUCED DISORDER
F11.950	OPIOID USE, UNSPECIFIED WITH OPIOID-INDUCED PSYCHOTIC DISORDER WITH DELUSIONS
F11.99	OPIOID USE, UNSPECIFIED WITH UNSPECIFIED OPIOID-INDUCED DISORDER
F12.122	CANNABIS ABUSE WITH INTOXICATION WITH PERCEPTUAL DISTURBANCE
F12.151	CANNABIS ABUSE WITH PSYCHOTIC DISORDER WITH HALLUCINATIONS
F12.19	CANNABIS ABUSE WITH UNSPECIFIED CANNABIS-INDUCED DISORDER
F12.288	CANNABIS DEPENDENCE WITH OTHER CANNABIS-INDUCED DISORDER

© 2020 Leung LB et al. *JAMA Network Open.*

F12.920	CANNABIS USE, UNSPECIFIED WITH INTOXICATION, UNCOMPLICATED
F12.921	CANNABIS USE, UNSPECIFIED WITH INTOXICATION DELIRIUM
F12.922	CANNABIS USE, UNSPECIFIED WITH INTOXICATION WITH PERCEPTUAL DISTURBANCE
F12.959	CANNABIS USE, UNSPECIFIED WITH PSYCHOTIC DISORDER, UNSPECIFIED
F13.14	SEDATIVE, HYPNOTIC OR ANXIOLYTIC ABUSE WITH SEDATIVE, HYPNOTIC OR ANXIOLYTIC-INDUCED MOOD DISORDER
F13.19	SEDATIVE, HYPNOTIC OR ANXIOLYTIC ABUSE WITH UNSPECIFIED SEDATIVE, HYPNOTIC OR ANXIOLYTIC-INDUCED DISORDER
F13.250	SEDATIVE, HYPNOTIC OR ANXIOLYTIC DEPENDENCE WITH SEDATIVE, HYPNOTIC OR ANXIOLYTIC-INDUCED PSYCHOTIC DISORDER WITH DELUSIONS
F13.282	SEDATIVE, HYPNOTIC OR ANXIOLYTIC DEPENDENCE WITH SEDATIVE, HYPNOTIC OR ANXIOLYTIC-INDUCED SLEEP DISORDER
F13.288	SEDATIVE, HYPNOTIC OR ANXIOLYTIC DEPENDENCE WITH OTHER SEDATIVE, HYPNOTIC OR ANXIOLYTIC-INDUCED DISORDER
F13.29	SEDATIVE, HYPNOTIC OR ANXIOLYTIC DEPENDENCE WITH UNSPECIFIED SEDATIVE, HYPNOTIC OR ANXIOLYTIC-INDUCED DISORDER
F13.930	SEDATIVE, HYPNOTIC OR ANXIOLYTIC USE, UNSPECIFIED WITH WITHDRAWAL, UNCOMPLICATED
F13.931	SEDATIVE, HYPNOTIC OR ANXIOLYTIC USE, UNSPECIFIED WITH WITHDRAWAL DELIRIUM
F13.932	SEDATIVE, HYPNOTIC OR ANXIOLYTIC USE, UNSPECIFIED WITH WITHDRAWAL WITH PERCEPTUAL DISTURBANCES
F13.951	SEDATIVE, HYPNOTIC OR ANXIOLYTIC USE, UNSPECIFIED WITH SEDATIVE, HYPNOTIC OR ANXIOLYTIC-INDUCED PSYCHOTIC DISORDER WITH HALLUCINATIONS
F13.980	SEDATIVE, HYPNOTIC OR ANXIOLYTIC USE, UNSPECIFIED WITH SEDATIVE, HYPNOTIC OR ANXIOLYTIC-INDUCED ANXIETY DISORDER
F14.10	COCAINE ABUSE, UNCOMPLICATED
F14.11	COCAINE ABUSE, IN REMISSION
F14.150	COCAINE ABUSE WITH COCAINE-INDUCED PSYCHOTIC DISORDER WITH DELUSIONS
F14.151	COCAINE ABUSE WITH COCAINE-INDUCED PSYCHOTIC DISORDER WITH HALLUCINATIONS
F14.159	COCAINE ABUSE WITH COCAINE-INDUCED PSYCHOTIC DISORDER, UNSPECIFIED
F14.180	COCAINE ABUSE WITH COCAINE-INDUCED ANXIETY DISORDER
F14.20	COCAINE DEPENDENCE, UNCOMPLICATED
F14.21	COCAINE DEPENDENCE, IN REMISSION
F14.222	COCAINE DEPENDENCE WITH INTOXICATION WITH PERCEPTUAL DISTURBANCE
F14.23	COCAINE DEPENDENCE WITH WITHDRAWAL
F14.251	COCAINE DEPENDENCE WITH COCAINE-INDUCED PSYCHOTIC DISORDER WITH HALLUCINATIONS
F14.259	COCAINE DEPENDENCE WITH COCAINE-INDUCED PSYCHOTIC DISORDER, UNSPECIFIED
F14.281	COCAINE DEPENDENCE WITH COCAINE-INDUCED SEXUAL DYSFUNCTION
F14.29	COCAINE DEPENDENCE WITH UNSPECIFIED COCAINE-INDUCED DISORDER
F14.90	COCAINE USE, UNSPECIFIED, UNCOMPLICATED
F14.929	COCAINE USE, UNSPECIFIED WITH INTOXICATION, UNSPECIFIED
F14.94	COCAINE USE, UNSPECIFIED WITH COCAINE-INDUCED MOOD DISORDER
F14.951	COCAINE USE, UNSPECIFIED WITH COCAINE-INDUCED PSYCHOTIC DISORDER WITH HALLUCINATIONS
F14.980	COCAINE USE, UNSPECIFIED WITH COCAINE-INDUCED ANXIETY DISORDER
F14.981	COCAINE USE, UNSPECIFIED WITH COCAINE-INDUCED SEXUAL DYSFUNCTION

© 2020 Leung LB et al. *JAMA Network Open.*

F14.982	COCAINE USE, UNSPECIFIED WITH COCAINE-INDUCED SLEEP DISORDER
F14.988	COCAINE USE, UNSPECIFIED WITH OTHER COCAINE-INDUCED DISORDER
F14.99	COCAINE USE, UNSPECIFIED WITH UNSPECIFIED COCAINE-INDUCED DISORDER
F10.10	ALCOHOL ABUSE, UNCOMPLICATED
F10.180	ALCOHOL ABUSE WITH ALCOHOL-INDUCED ANXIETY DISORDER
F10.230	ALCOHOL DEPENDENCE WITH WITHDRAWAL, UNCOMPLICATED
F10.251	ALCOHOL DEPENDENCE WITH ALCOHOL-INDUCED PSYCHOTIC DISORDER WITH HALLUCINATIONS
F10.920	ALCOHOL USE, UNSPECIFIED WITH INTOXICATION, UNCOMPLICATED
F10.921	ALCOHOL USE, UNSPECIFIED WITH INTOXICATION DELIRIUM
F10.929	ALCOHOL USE, UNSPECIFIED WITH INTOXICATION, UNSPECIFIED
F10.951	ALCOHOL USE, UNSPECIFIED WITH ALCOHOL-INDUCED PSYCHOTIC DISORDER WITH HALLUCINATIONS
F10.959	ALCOHOL USE, UNSPECIFIED WITH ALCOHOL-INDUCED PSYCHOTIC DISORDER, UNSPECIFIED
F10.96	ALCOHOL USE, UNSPECIFIED WITH ALCOHOL-INDUCED PERSISTING AMNESTIC DISORDER
F11.182	OPIOID ABUSE WITH OPIOID-INDUCED SLEEP DISORDER
F11.188	OPIOID ABUSE WITH OTHER OPIOID-INDUCED DISORDER
F11.19	OPIOID ABUSE WITH UNSPECIFIED OPIOID-INDUCED DISORDER
F11.220	OPIOID DEPENDENCE WITH INTOXICATION, UNCOMPLICATED
F11.24	OPIOID DEPENDENCE WITH OPIOID-INDUCED MOOD DISORDER
F11.281	OPIOID DEPENDENCE WITH OPIOID-INDUCED SEXUAL DYSFUNCTION
F11.288	OPIOID DEPENDENCE WITH OTHER OPIOID-INDUCED DISORDER
F11.922	OPIOID USE, UNSPECIFIED WITH INTOXICATION WITH PERCEPTUAL DISTURBANCE
F11.951	OPIOID USE, UNSPECIFIED WITH OPIOID-INDUCED PSYCHOTIC DISORDER WITH HALLUCINATIONS
F11.959	OPIOID USE, UNSPECIFIED WITH OPIOID-INDUCED PSYCHOTIC DISORDER, UNSPECIFIED
F12.120	CANNABIS ABUSE WITH INTOXICATION, UNCOMPLICATED
F12.129	CANNABIS ABUSE WITH INTOXICATION, UNSPECIFIED
F12.20	CANNABIS DEPENDENCE, UNCOMPLICATED
F12.21	CANNABIS DEPENDENCE, IN REMISSION
F12.220	CANNABIS DEPENDENCE WITH INTOXICATION, UNCOMPLICATED
F12.221	CANNABIS DEPENDENCE WITH INTOXICATION DELIRIUM
F12.222	CANNABIS DEPENDENCE WITH INTOXICATION WITH PERCEPTUAL DISTURBANCE
F12.229	CANNABIS DEPENDENCE WITH INTOXICATION, UNSPECIFIED
F12.259	CANNABIS DEPENDENCE WITH PSYCHOTIC DISORDER, UNSPECIFIED
F12.29	CANNABIS DEPENDENCE WITH UNSPECIFIED CANNABIS-INDUCED DISORDER
F12.93	CANNABIS USE, UNSPECIFIED WITH WITHDRAWAL
F12.951	CANNABIS USE, UNSPECIFIED WITH PSYCHOTIC DISORDER WITH HALLUCINATIONS
F12.988	CANNABIS USE, UNSPECIFIED WITH OTHER CANNABIS-INDUCED DISORDER
F13.120	SEDATIVE, HYPNOTIC OR ANXIOLYTIC ABUSE WITH INTOXICATION, UNCOMPLICATED
F13.159	SEDATIVE, HYPNOTIC OR ANXIOLYTIC ABUSE WITH SEDATIVE, HYPNOTIC OR ANXIOLYTIC-INDUCED PSYCHOTIC DISORDER, UNSPECIFIED
F13.180	SEDATIVE, HYPNOTIC OR ANXIOLYTIC ABUSE WITH SEDATIVE, HYPNOTIC OR ANXIOLYTIC-INDUCED ANXIETY DISORDER
F13.220	SEDATIVE, HYPNOTIC OR ANXIOLYTIC DEPENDENCE WITH INTOXICATION, UNCOMPLICATED

© 2020 Leung LB et al. *JAMA Network Open.*

F13.259	SEDATIVE, HYPNOTIC OR ANXIOLYTIC DEPENDENCE WITH SEDATIVE, HYPNOTIC OR ANXIOLYTIC-INDUCED PSYCHOTIC DISORDER, UNSPECIFIED
F13.26	SEDATIVE, HYPNOTIC OR ANXIOLYTIC DEPENDENCE WITH SEDATIVE, HYPNOTIC OR ANXIOLYTIC-INDUCED PERSISTING AMNESTIC DISORDER
F13.920	SEDATIVE, HYPNOTIC OR ANXIOLYTIC USE, UNSPECIFIED WITH INTOXICATION, UNCOMPLICATED
F13.939	SEDATIVE, HYPNOTIC OR ANXIOLYTIC USE, UNSPECIFIED WITH WITHDRAWAL, UNSPECIFIED
F13.988	SEDATIVE, HYPNOTIC OR ANXIOLYTIC USE, UNSPECIFIED WITH OTHER SEDATIVE, HYPNOTIC OR ANXIOLYTIC-INDUCED DISORDER
F13.99	SEDATIVE, HYPNOTIC OR ANXIOLYTIC USE, UNSPECIFIED WITH UNSPECIFIED SEDATIVE, HYPNOTIC OR ANXIOLYTIC-INDUCED DISORDER
F14.120	COCAINE ABUSE WITH INTOXICATION, UNCOMPLICATED
F14.121	COCAINE ABUSE WITH INTOXICATION WITH DELIRIUM
F14.14	COCAINE ABUSE WITH COCAINE-INDUCED MOOD DISORDER
F14.221	COCAINE DEPENDENCE WITH INTOXICATION DELIRIUM
F14.280	COCAINE DEPENDENCE WITH COCAINE-INDUCED ANXIETY DISORDER
F14.288	COCAINE DEPENDENCE WITH OTHER COCAINE-INDUCED DISORDER
F14.922	COCAINE USE, UNSPECIFIED WITH INTOXICATION WITH PERCEPTUAL DISTURBANCE
F14.950	COCAINE USE, UNSPECIFIED WITH COCAINE-INDUCED PSYCHOTIC DISORDER WITH DELUSIONS
F14.959	COCAINE USE, UNSPECIFIED WITH COCAINE-INDUCED PSYCHOTIC DISORDER, UNSPECIFIED
F19.10	OTHER PSYCHOACTIVE SUBSTANCE ABUSE, UNCOMPLICATED
F19.11	OTHER PSYCHOACTIVE SUBSTANCE ABUSE, IN REMISSION
F19.120	OTHER PSYCHOACTIVE SUBSTANCE ABUSE WITH INTOXICATION, UNCOMPLICATED
F19.121	OTHER PSYCHOACTIVE SUBSTANCE ABUSE WITH INTOXICATION DELIRIUM
F19.122	OTHER PSYCHOACTIVE SUBSTANCE ABUSE WITH INTOXICATION WITH PERCEPTUAL DISTURBANCES
F19.129	OTHER PSYCHOACTIVE SUBSTANCE ABUSE WITH INTOXICATION, UNSPECIFIED
F19.14	OTHER PSYCHOACTIVE SUBSTANCE ABUSE WITH PSYCHOACTIVE SUBSTANCE-INDUCED MOOD DISORDER
F19.150	OTHER PSYCHOACTIVE SUBSTANCE ABUSE WITH PSYCHOACTIVE SUBSTANCE-INDUCED PSYCHOTIC DISORDER WITH DELUSIONS
F19.151	OTHER PSYCHOACTIVE SUBSTANCE ABUSE WITH PSYCHOACTIVE SUBSTANCE-INDUCED PSYCHOTIC DISORDER WITH HALLUCINATIONS
F19.159	OTHER PSYCHOACTIVE SUBSTANCE ABUSE WITH PSYCHOACTIVE SUBSTANCE-INDUCED PSYCHOTIC DISORDER, UNSPECIFIED
F19.16	OTHER PSYCHOACTIVE SUBSTANCE ABUSE WITH PSYCHOACTIVE SUBSTANCE-INDUCED PERSISTING AMNESTIC DISORDER
F19.17	OTHER PSYCHOACTIVE SUBSTANCE ABUSE WITH PSYCHOACTIVE SUBSTANCE-INDUCED PERSISTING DEMENTIA
F19.180	OTHER PSYCHOACTIVE SUBSTANCE ABUSE WITH PSYCHOACTIVE SUBSTANCE-INDUCED ANXIETY DISORDER
F19.181	OTHER PSYCHOACTIVE SUBSTANCE ABUSE WITH PSYCHOACTIVE SUBSTANCE-INDUCED SEXUAL DYSFUNCTION
F19.182	OTHER PSYCHOACTIVE SUBSTANCE ABUSE WITH PSYCHOACTIVE SUBSTANCE-INDUCED SLEEP DISORDER
F19.188	OTHER PSYCHOACTIVE SUBSTANCE ABUSE WITH OTHER PSYCHOACTIVE SUBSTANCE-INDUCED DISORDER
F19.19	OTHER PSYCHOACTIVE SUBSTANCE ABUSE WITH UNSPECIFIED PSYCHOACTIVE SUBSTANCE-INDUCED DISORDER
F19.20	OTHER PSYCHOACTIVE SUBSTANCE DEPENDENCE, UNCOMPLICATED
F19.21	OTHER PSYCHOACTIVE SUBSTANCE DEPENDENCE, IN REMISSION
F19.220	OTHER PSYCHOACTIVE SUBSTANCE DEPENDENCE WITH INTOXICATION, UNCOMPLICATED

© 2020 Leung LB et al. *JAMA Network Open.*

F19.221	OTHER PSYCHOACTIVE SUBSTANCE DEPENDENCE WITH INTOXICATION DELIRIUM
F19.222	OTHER PSYCHOACTIVE SUBSTANCE DEPENDENCE WITH INTOXICATION WITH PERCEPTUAL DISTURBANCE
F19.229	OTHER PSYCHOACTIVE SUBSTANCE DEPENDENCE WITH INTOXICATION, UNSPECIFIED
F19.230	OTHER PSYCHOACTIVE SUBSTANCE DEPENDENCE WITH WITHDRAWAL, UNCOMPLICATED
F19.231	OTHER PSYCHOACTIVE SUBSTANCE DEPENDENCE WITH WITHDRAWAL DELIRIUM
F19.232	OTHER PSYCHOACTIVE SUBSTANCE DEPENDENCE WITH WITHDRAWAL WITH PERCEPTUAL DISTURBANCE
F19.239	OTHER PSYCHOACTIVE SUBSTANCE DEPENDENCE WITH WITHDRAWAL, UNSPECIFIED
F19.24	OTHER PSYCHOACTIVE SUBSTANCE DEPENDENCE WITH PSYCHOACTIVE SUBSTANCE-INDUCED MOOD DISORDER
F19.250	OTHER PSYCHOACTIVE SUBSTANCE DEPENDENCE WITH PSYCHOACTIVE SUBSTANCE-INDUCED PSYCHOTIC DISORDER WITH DELUSIONS
F19.251	OTHER PSYCHOACTIVE SUBSTANCE DEPENDENCE WITH PSYCHOACTIVE SUBSTANCE-INDUCED PSYCHOTIC DISORDER WITH HALLUCINATIONS
F19.259	OTHER PSYCHOACTIVE SUBSTANCE DEPENDENCE WITH PSYCHOACTIVE SUBSTANCE-INDUCED PSYCHOTIC DISORDER, UNSPECIFIED
F19.26	OTHER PSYCHOACTIVE SUBSTANCE DEPENDENCE WITH PSYCHOACTIVE SUBSTANCE-INDUCED PERSISTING AMNESTIC DISORDER
F19.27	OTHER PSYCHOACTIVE SUBSTANCE DEPENDENCE WITH PSYCHOACTIVE SUBSTANCE-INDUCED PERSISTING DEMENTIA
F19.280	OTHER PSYCHOACTIVE SUBSTANCE DEPENDENCE WITH PSYCHOACTIVE SUBSTANCE-INDUCED ANXIETY DISORDER
F19.281	OTHER PSYCHOACTIVE SUBSTANCE DEPENDENCE WITH PSYCHOACTIVE SUBSTANCE-INDUCED SEXUAL DYSFUNCTION
F19.282	OTHER PSYCHOACTIVE SUBSTANCE DEPENDENCE WITH PSYCHOACTIVE SUBSTANCE-INDUCED SLEEP DISORDER
F19.288	OTHER PSYCHOACTIVE SUBSTANCE DEPENDENCE WITH OTHER PSYCHOACTIVE SUBSTANCE-INDUCED DISORDER
F19.29	OTHER PSYCHOACTIVE SUBSTANCE DEPENDENCE WITH UNSPECIFIED PSYCHOACTIVE SUBSTANCE-INDUCED DISORDER
F19.90	OTHER PSYCHOACTIVE SUBSTANCE USE, UNSPECIFIED, UNCOMPLICATED
F19.920	OTHER PSYCHOACTIVE SUBSTANCE USE, UNSPECIFIED WITH INTOXICATION, UNCOMPLICATED
F19.921	OTHER PSYCHOACTIVE SUBSTANCE USE, UNSPECIFIED WITH INTOXICATION WITH DELIRIUM
F19.922	OTHER PSYCHOACTIVE SUBSTANCE USE, UNSPECIFIED WITH INTOXICATION WITH PERCEPTUAL DISTURBANCE
F19.929	OTHER PSYCHOACTIVE SUBSTANCE USE, UNSPECIFIED WITH INTOXICATION, UNSPECIFIED
F19.930	OTHER PSYCHOACTIVE SUBSTANCE USE, UNSPECIFIED WITH WITHDRAWAL, UNCOMPLICATED
F19.931	OTHER PSYCHOACTIVE SUBSTANCE USE, UNSPECIFIED WITH WITHDRAWAL DELIRIUM
F19.932	OTHER PSYCHOACTIVE SUBSTANCE USE, UNSPECIFIED WITH WITHDRAWAL WITH PERCEPTUAL DISTURBANCE
F19.939	OTHER PSYCHOACTIVE SUBSTANCE USE, UNSPECIFIED WITH WITHDRAWAL, UNSPECIFIED
F19.94	OTHER PSYCHOACTIVE SUBSTANCE USE, UNSPECIFIED WITH PSYCHOACTIVE SUBSTANCE-INDUCED MOOD DISORDER
F19.950	OTHER PSYCHOACTIVE SUBSTANCE USE, UNSPECIFIED WITH PSYCHOACTIVE SUBSTANCE-INDUCED PSYCHOTIC DISORDER WITH DELUSIONS
F19.951	OTHER PSYCHOACTIVE SUBSTANCE USE, UNSPECIFIED WITH PSYCHOACTIVE SUBSTANCE-INDUCED PSYCHOTIC DISORDER WITH HALLUCINATIONS
F19.959	OTHER PSYCHOACTIVE SUBSTANCE USE, UNSPECIFIED WITH PSYCHOACTIVE SUBSTANCE-INDUCED PSYCHOTIC DISORDER, UNSPECIFIED

© 2020 Leung LB et al. *JAMA Network Open.*

F19.96	OTHER PSYCHOACTIVE SUBSTANCE USE, UNSPECIFIED WITH PSYCHOACTIVE SUBSTANCE-INDUCED PERSISTING AMNESTIC DISORDER
F19.97	OTHER PSYCHOACTIVE SUBSTANCE USE, UNSPECIFIED WITH PSYCHOACTIVE SUBSTANCE-INDUCED PERSISTING DEMENTIA
F19.980	OTHER PSYCHOACTIVE SUBSTANCE USE, UNSPECIFIED WITH PSYCHOACTIVE SUBSTANCE-INDUCED ANXIETY DISORDER
F19.981	OTHER PSYCHOACTIVE SUBSTANCE USE, UNSPECIFIED WITH PSYCHOACTIVE SUBSTANCE-INDUCED SEXUAL DYSFUNCTION
F19.982	OTHER PSYCHOACTIVE SUBSTANCE USE, UNSPECIFIED WITH PSYCHOACTIVE SUBSTANCE-INDUCED SLEEP DISORDER
F19.988	OTHER PSYCHOACTIVE SUBSTANCE USE, UNSPECIFIED WITH OTHER PSYCHOACTIVE SUBSTANCE-INDUCED DISORDER
F19.99	OTHER PSYCHOACTIVE SUBSTANCE USE, UNSPECIFIED WITH UNSPECIFIED PSYCHOACTIVE SUBSTANCE-INDUCED DISORDER

© 2020 Leung LB et al. *JAMA Network Open.*
